# TRMT112 drives a tumor growth and metastasis-promoting program in triple-negative breast cancer

**DOI:** 10.1038/s41418-025-01643-z

**Published:** 2026-01-08

**Authors:** Amr R. Elhamamsy, Brandon J. Metge, Mohamed H. Elbahoty, Bhavyasree Papineni, Heba Allah M. Alsheikh, Dongquan Chen, Rajeev S. Samant, Lalita A. Shevde

**Affiliations:** 1 Department of Pathology, University of Alabama at Birmingham, Birmingham, AL USA; 2 Division of General Internal Medicine and Population Science, Department of Medicine, University of Alabama at Birmingham, Birmingham, AL USA; 3 O’Neal Comprehensive Cancer Center, University of Alabama at Birmingham, Birmingham, AL USA

**Keywords:** Tumour biomarkers, Metastasis

## Abstract

Ribosomal RNA Modifying Proteins (RRMPs) are integral to ribosome biogenesis, executing post-transcriptional modifications that influence translation fidelity and efficiency. Dysregulation of RRMPs has been increasingly implicated in cancer progression, yet their collective role across malignancies remains largely unexplored. Here, we performed a multi-omics analysis of 22 RRMPs across diverse cancer types using The Cancer Genome Atlas, the Molecular Taxonomy of Breast Cancer International Consortium, and additional high-throughput datasets. Our analysis revealed widespread genomic alterations and transcriptional dysregulation of RRMPs across malignancies, with distinct expression patterns in breast cancer subtypes. Notably, Triple-Negative Breast Cancer (TNBC) exhibited the highest RRMPs enrichment, which correlated with increased genomic instability including elevated tumor mutational burden and aneuploidy scores, and poor survival outcomes. Among the RRMPs, tRNA methyltransferase activator subunit 11-2 (TRMT112) emerged as a key regulator of tumor progression. Functional assays demonstrated that TRMT112 knockdown in TNBC cells significantly reduced proliferation, migration, invasion, and metastatic potential, whereas its overexpression enhanced these tumorigenic properties. Polysome profiling and RNA sequencing of actively translated transcripts revealed that TRMT112 reprograms the translational landscape by promoting pro-metastatic and stromal remodeling pathways while suppressing immune-related processes. In vivo studies using an orthotopic breast cancer model further confirmed that TRMT112 depletion impairs tumor growth and reduces metastatic burden. Collectively, our findings establish RRMPs as critical modulators of cancer progression and identify TRMT112 as a key driver of aggressive phenotypes in TNBC. The dysregulation of TRMT112 across breast cancer subtypes highlights its potential as both a prognostic biomarker and a therapeutic target. These insights provide a mechanistic foundation for future interventions aimed at targeting TRMT112-driven translational programs in aggressive breast cancer.

## Introduction

The landscape of cancer biology is markedly influenced by dynamic alterations in ribosomal RNA (rRNA), which plays a pivotal role in cellular function and organismal physiology [1, 2]. Among the multifarious factors contributing to the complexity of cancer, Ribosomal RNA Modifying Proteins (RRMPs) are gaining prominence due to their intricate involvement in the modification of rRNA, essential for ribosome biogenesis and function [3–5]. However, despite their fundamental roles, little is known about the specific contributions of RRMPs to cancer biology, and their potential as therapeutic targets remains underexplored.

RRMPs are not mere participants in the translational machinery but are central to the post-transcriptional modifications that govern the fate of rRNA, impacting everything from peptide bond formation to the fidelity of protein synthesis [6, 7]. Dysregulation of RRMPs is increasingly recognized as a determinant of tumorigenicity, influencing cancer onset, progression, and patient outcomes [8–10]. Genetic alterations in RRMPs, ranging from mutations to copy number variations, have been implicated in various cancers [11, 12]. Yet, the full spectrum of RRMPs alteration across cancer types and their functional consequences remain inadequately understood, necessitating a focused investigation into these proteins.

Through functional dependency screening our study identified that tRNA methyltransferase activator subunit 11-2 (TRMT112) is essential for breast cancer cell survival, with its expression significantly elevated in tumors and metastatic lesions compared to normal tissue. Notably, TRMT112 was highly enriched in aggressive breast cancer subtypes, particularly triple-negative breast cancer (TNBC), where its overexpression is associated with poor clinical outcomes, including reduced disease-specific survival. TRMT112 functions as an activator of methyltransferases involved in rRNA modification [13, 14], specifically catalyzing methylation at positions 1639 and 1832 of 18S rRNA, which are critical for ribosome assembly and function [15, 16].

Beyond rRNA modification, TRMT112 serves as a multifunctional hub protein that stabilizes and activates a diverse set of methyltransferases. These include enzymes involved in tRNA modification (e.g., TRMT11, ALKBH8, THUMPD2/3), protein and histone methylation, and even mRNA methylation, thereby linking TRMT112 to multiple aspects of RNA metabolism, genome stability, and cellular stress responses [13, 17–22]. This versatility underscores TRMT112’s potential to influence cancer-relevant pathways beyond ribosome biogenesis, including translational reprogramming, adaptation to metabolic stress, and invasive phenotypes. Despite this functional diversity, TRMT112’s role in cancer remains poorly understood.

In this study we systematically investigated the alterations in RRMPs, with a specific focus on TRMT112, across different breast cancer subtypes and elucidated its role in tumor growth, invasion, and metastasis. Breast cancer, the most common malignancy among women worldwide, exhibits significant heterogeneity across its subtypes - Luminal A, Luminal B, HER2-enriched, and TNBC [2, 23, 24]. Furthermore, exacerbated by the absence of targeted therapies, the metastatic propensity of TNBC remains a challenge [25–27]. In this study we present evidence to support that each subtype exhibits distinct RRMPs expression patterns and demonstrate that TNBC supports elevated expression of TRMT112. We comprehensively evaluated the role of TRMT112 in breast cancer utilizing public databases to delineate RRMPs mutations and expression levels, especially TRMT112, across different subtypes of breast cancer and elucidate its functional relevance in metastasis. By integrating data from The Cancer Genome Atlas (TCGA), METABRIC, and other high-throughput datasets, our study provides a detailed portrait of TRMT112 alterations and their implications in breast cancer. Our findings suggest that TRMT112 plays a pivotal role in TNBC, highlighting its potential as a prognostic biomarker and/or a therapeutic target, and provide a mechanistic foundation for future interventions aimed at targeting TRMT112-driven translational programs in aggressive breast cancer.

## Results

### Alterations in RRMPs are pervasive across cancer types and linked to poor survival outcomes

**Fig. 1:**
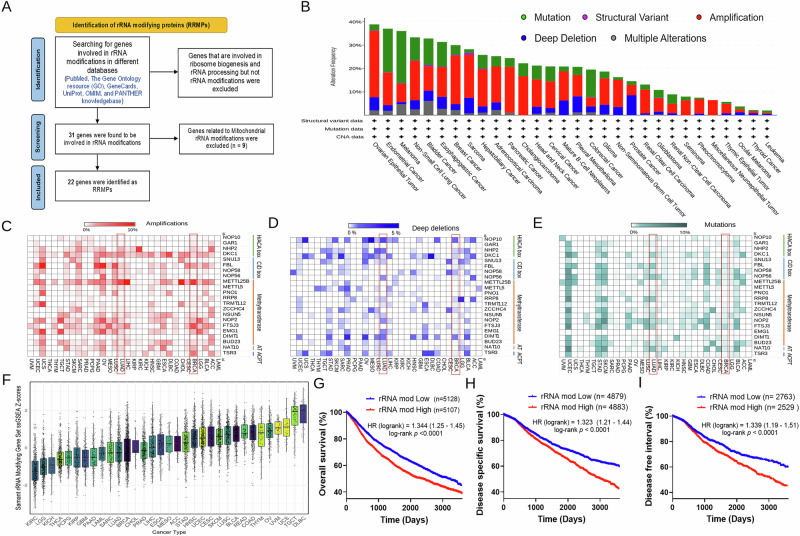
RRMPs alterations are pervasive across cancer types and linked to poor survival outcomes. **A** A schematic workflow outlines the multi-step process to identify genes involved in rRNA modifications, focusing on those relevant to ribosomal biogenesis and cancer while excluding mitochondrial-related genes. **B** A bar graph displays the frequency of RRMPs alterations, including structural variants (green), mutations (red), and copy number alterations (blue), across various cancer types using cBioPortal (TCGA) data. The high prevalence of these alterations suggests RRMPs’ potential role in tumorigenesis and therapeutic targeting. Heatmaps illustrate the distribution of RRMPs alterations: amplifications (**C**), deep deletions (**D**), and mutations (**E**) across multiple cancers. Each row represents an RRMP categorized into H/ACA box, C/D box, or methyltransferases, while columns denote specific cancer types. The intensity of red reflects the frequency of alterations, with notable enrichment in cancers such as BRCA and LUAD. **F** A boxplot shows the enrichment of a 22-gene RRMPs signature, calculated using single-sample Gene Set Enrichment Analysis (ssGSEA), across TCGA cancer types. Higher enrichment scores are observed in aggressive cancers like DLBC and UCS, whereas cancers like KIRC and LGG display lower enrichment, highlighting subtype-specific RRMPs involvement. **G** Kaplan–Meier analysis reveals that patients with high RRMPs expression levels (red) have significantly shorter 10-year overall survival compared to those with low expression (blue) across various cancers analyzed using TCGA data (HR = 1.344, *p* < 0.0001). **H** 10-year disease-specific survival analysis shows that elevated RRMPs levels (red) are associated with worse prognosis relative to low-expressing patients (blue) across different cancers in TCGA data (HR = 1.323, *p* < 0.0001). **I** A Kaplan–Meier plot illustrates that patients with high RRMPs expression (red) have significantly reduced 10-year disease-free survival compared to those with lower expression (blue) across different cancers in TCGA data (HR = 1.338, *p* < 0.0001).

To explore the functional impacts of RRMPs in various cancers, we defined a specific 22-gene RRMPs signature. This signature was established based on a thorough literature review and database searches that identified genes consistently implicated in rRNA modification and associated with critical outcomes in cancer biology. The selection focused on genes directly involved in the enzymatic processes altering rRNA, which are essential for proper ribosome function and thus influence protein synthesis crucial for cell growth and division (Fig. 1A), while excluding proteins associated with mitochondrial ribosome-related modifications. To elucidate the role of RRMPs in cancer, we investigated TCGA data. This analysis revealed a significant frequency of genomic alterations (i.e., amplifications, mutations, and deletions) in RRMPs genes across a variety of cancer types (Fig. 1B). Further, detailed heatmaps illustrated the specific distribution and frequency of RRMPs alterations, categorizing them as amplifications, deep deletions, and mutations (Fig. 1C–E). This detailed visualization showed pronounced variability in alteration patterns across different cancers, notably in breast cancer (BRCA) and lung adenocarcinoma (LUAD). We assessed a 22-gene RRMPs signature enrichment through single-sample Gene Set Enrichment Analysis across all sampled cancers (Fig. 1F). Notable differences were observed in the enrichment of this signature among various cancers, with types such as diffuse large B-cell lymphoma (DLBC) and uterine carcinosarcoma (UCS) displaying notably higher enrichment, while renal clear cell carcinoma (KIRC) and lower grade glioma (LGG) showed reduced enrichment.

Kaplan–Meier analyses across 32 types of cancers from TCGA data demonstrated that high RRMPs levels significantly correlated with poorer overall survival (Fig. 1G), disease-specific survival (Fig. 1H), and disease-free survival (Fig. 1I), with hazard ratios of 1.344, 1.323, and 1.338, respectively, all achieving highly significant log-rank *p*-values (<0.0001). These findings highlight RRMPs as potential prognostic biomarkers in cancer.

### Expression of RRMPs distinguishes tumors from normal tissue and is linked to poor survival and metastasis in breast cancer

We further explored the genetic landscape of RRMPs in breast cancer. Analysis of METABRIC and TCGA BRCA data revealed frequent genetic alterations in RRMPs, particularly amplifications, which were prevalent across various breast cancer subtypes (Supplementary Fig. S1A). Notably, the breast cancer basal subtype exhibited a significantly higher prevalence of RRMPs alterations compared to other subtypes (Supplementary Fig. S1B), suggesting a link between the alterations of RRMPs and this aggressive breast cancer subtype. Next, we examined the association between the alterations of RRMPs and genomic instability in breast cancer. Alterations of RRMPs were significantly correlated with increased tumor mutational burden, higher aneuploidy scores, and a greater fraction of genome altered (Supplementary Fig. S1C–E). These associations indicate a potential role of RRMPs in driving genomic instability and tumorigenesis.

**Fig. 2:**
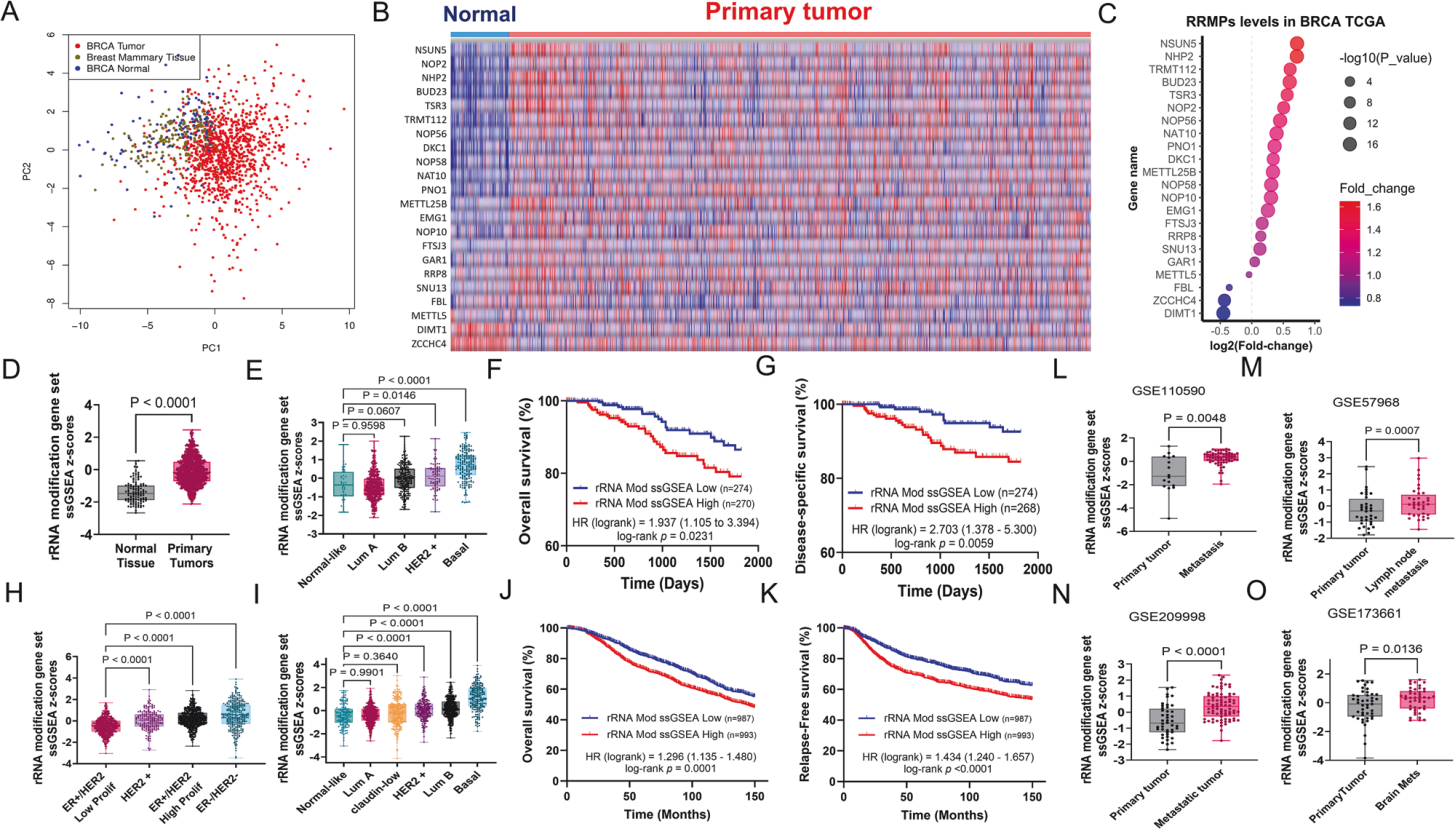
Expression of RRMPs distinguishes tumors from normal tissue and is linked to poor survival and metastasis in breast cancer. **A** A 2D PCA plot reveals distinct clustering of breast cancer (BRCA) tumors, normal breast tissues from BRCA patients, and normal mammary tissues from the GTEx database, highlighting significant differences in RRMPs expression profiles across these tissue types. **B** A heatmap comparing RRMPs expression in BRCA tumor and matched normal tissues (TCGA cohort) shows widespread dysregulation, with many RRMPs markedly upregulated in tumors. **C** A bubble plot presents the log2 fold-change of RRMPs between tumor and normal tissues, with significant upregulation of key RRMPs, including NSUN5 and TRMT112, suggesting their potential roles in tumor progression. **D** A boxplot comparing single-sample Gene Set Enrichment Analysis (ssGSEA) scores demonstrates significantly higher RRMPs signature enrichment in primary breast tumors versus normal tissues (*p* < 0.0001), indicating the association of RRMPs activity with tumorigenesis. **E** A boxplot reveals significant variation in RRMPs signature enrichment across breast cancer subtypes using TCGA data, with the Basal subtype showing the highest and Luminal A the lowest enrichment (*p* < 0.0001). **F** Kaplan–Meier curve shows that TCGA BRCA patients with high RRMPs signature enrichment (red) have significantly reduced five-year overall survival compared to those with low enrichment (blue) (HR = 1.937, *p* = 0.0231). **G** Kaplan–Meier curve illustrates that high RRMP signature enrichment (red) correlate with poorer disease-specific survival (HR = 2.703, *p* = 0.0059) in TCGA BRCA patients, reinforcing the prognostic value of RRMPs activity. **H** A boxplot shows the ssGSEA scores of the RRMPs signature across different breast cancer subtypes classified by the 3-Gene classifier using METABRIC data, with the ER-/HER2- subtype displaying the highest scores (*p* < 0.0001). **I** A boxplot illustrates the ssGSEA scores of the RRMPs signature across breast cancer subtypes classified by the PAM50 and Claudin-low classifications using METABRIC data. RRMPs enrichment is significantly elevated in Basal subtype compared to Luminal A and Normal-like subtypes (*p* < 0.0001). **J** Kaplan–Meier survival curve shows poor overall survival in breast cancer patients with high RRMPs signature enrichment (red) compared to patients with low RRMPs signature (blue) using METABRIC data. (HR = 1.296, *p* = 0.0001). **K** Kaplan–Meier curve illustrates that high RRMPs signature enrichment (red) correlate with poorer relapse-free survival compared to those with low enrichment in METABRIC data (blue) (HR = 1.434, *p* < 0.0001). **L** A boxplot shows significantly higher RRMPs signature enrichment in metastatic samples compared to matched primary tumors (*p* = 0.0048) using the GSE110590 dataset comprising matched primary tumors and multiple metastases from 16 patients, indicating RRMPs involvement in metastatic progression. **M** A boxplot illustrates higher ssGSEA scores of the RRMPs signature in lymph node metastases compared to primary breast tumors (*p* = 0.0007) using the GSE57968 dataset comprising 36 paired primary and regional metastatic breast cancer samples, underscoring the potential role of RRMPs in regional spread. **N** A boxplot shows that the ssGSEA scores of the RRMPs signature in metastatic tumors (*n* = 79) are significantly higher (*p* < 0.0001) than primary breast tumors (*n* = 44) using the GSE209998 dataset. **O** A boxplot shows RRMPs signature enrichment in brain metastatic tumors (*n* = 45) is markedly increased compared to matched primary breast tumors (*n* = 45) using data from the GSE173661 dataset, highlighting a potential role of RRMPs in brain metastatic colonization.

To explore the gene expression levels of RRMPs in breast cancer, we employed a 2D principal component analysis to compare the expression profiles of RRMPs across breast tumors, matched normal breast tissues from cancer patients, and normal mammary tissues from the GTEx database (Fig. 2A). This analysis revealed distinct clustering patterns, with breast tumors forming a separate cluster from matched normal breast tissues, and GTEx mammary tissues indicating that RRMPs are differentially expressed across these tissue types. Further examination of RRMPs expression in the TCGA breast cancer cohort showed a significant upregulation and enrichment in tumor tissue compared to normal tissues (Fig. 2B and Supplementary Fig. S1F, G). The heatmap highlights the widespread dysregulation of RRMPs in breast cancer, with certain RRMPs, such as NSUN5, NHP2 and TRMT112, exhibiting marked upregulation in tumor tissues compared to normal tissues (Fig. 2C).

To assess the functional relevance of RRMPs in breast cancer, we performed single-sample Gene Set Enrichment Analysis (ssGSEA) using the TCGA-BRCA dataset. The results demonstrated a significantly higher enrichment of the RRMPs signature in primary tumors compared to normal tissues (*p* < 0.0001), underscoring the strong association between RRMPs expression levels and breast cancer development (Fig. 2D). Additionally, the expression signature of RRMPs showed differential enrichment across various breast cancer subtypes, with the highest levels observed in the basal subtype and the lowest in luminal A (*p* < 0.0001), indicating subtype-specific roles for RRMPs (Fig. 2E).

Kaplan-Meier survival analyses revealed that higher enrichment of RRMPs signature is associated with poorer clinical outcomes. In the TCGA-BRCA cohort, patients with high RRMPs ssGSEA scores had significantly worse five-year overall survival (HR = 1.937, *p* = 0.0231) and disease-specific survival (HR = 2.703, *p* = 0.0059) compared to patients with lower scores (Fig. 2F, G). These findings were further validated in the METABRIC cohort, where elevated enrichment of RRMPs signature was predominantly associated with the ER-/HER2- and basal subtypes (Fig. 2H, I). Additionally, higher RRMPs enrichment correlated with poorer clinical outcomes, including significantly reduced overall survival (HR = 1.298, *p* = 0.0001) and relapse-free survival (HR = 1.434, *p* < 0.0001) (Fig. 2J, K), reinforcing its potential prognostic significance in breast cancer. We analyzed data from multiple datasets to explore the role of RRMPs in metastasis. In the GSE110590 dataset, enrichment of RRMPs signature was significantly higher in metastatic samples compared to primary tumors (*p* = 0.0048), suggesting a potential role of RRMPs in metastatic progression (Fig. 2L). Similar trends were observed in the GSE57968, GSE209998 and GSE173661 datasets, where RRMPs signature enrichment was elevated in lymph node, distant metastases and brain metastases, respectively, compared to primary breast tumors (Fig. 2M–O). Cumulatively, the data demonstrates that RRMPs are dysregulated in breast cancer, possibly contributing to genomic instability, poor survival, and metastasis, especially in aggressive subtypes.

### Elevated RRMPs signature is linked to genomic instability and tumor aggressiveness in breast cancer

To further elucidate the functional implications of RRMPs in breast cancer, we analyzed the correlation between enrichment of RRMPs signature and various tumor attributes using TCGA-BRCA data. We found a significant positive correlation between RRMPs ssGSEA scores and the homologous recombination deficiency (HRD) score (Pearson’s r = 0.4687, *p* < 0.0001), suggesting a link between RRMPs activity and impaired DNA repair mechanisms (Supplementary Fig. S2A). Additionally, higher RRMPs enrichment was associated with increased tumor stemness (Pearson’s r = 0.5765, *p* < 0.0001) and reduced stromal content (Pearson’s r = -0.3957, *p* < 0.0001), as reflected by the Stemness and Stromal Scores, respectively (Supplementary Fig. S2B, C). A modest negative correlation was also observed between RRMPs enrichment and the ESTIMATE Score, indicating reduced immune and stromal content in tumors with high RRMPs activity (Supplementary Fig. S2D). We also examined RRMPs signature enrichment across different Nottingham histological grades in the GSE202203 dataset. Our analysis revealed a significant increase in RRMPs enrichment with higher histological grades, indicating that RRMPs are more active in aggressive breast cancer phenotypes (Supplementary Fig. S2E). This trend was further supported by an analysis of RRMPs enrichment across various PAM50 breast cancer subtypes, where the basal subtype exhibited the highest RRMPs activity (Supplementary Fig. S2F).

To explore the regulatory dynamics of RRMPs in breast cancer, we examined their co-expression patterns across normal, primary tumor, and metastatic tissues using similarity matrices. Given that rRNA modifications can occur concurrently on different rRNA species, analyzing co-expression provided important insight into the coordinated regulation of RRMPs and their potential role in cancer progression. In normal tissues, RRMPs showed strong positive correlations, indicating a tightly regulated network (Supplementary Fig. S3A). This coordination weakened in tumors, suggesting dysregulation of rRNA modifications (Supplementary Fig. S3B), and became more disrupted in metastases (Supplementary Fig. S3C), where both positive and negative correlations emerged, reflecting a complex reprogramming of RRMPs interactions. Notably, TRMT112 exhibited significant shifts in correlation with its functional partners, BUD23 and METTL5, across disease states, highlighting its potential role in tumor progression and metastasis.

We analyzed scRNA-seq data from GSE176078 using the Single Cell Portal to explore the context-specific expression of RRMPs at the single-cell level. This revealed distinct expression patterns of RRMPs across breast cancer subtypes and microenvironmental compartments (Supplementary Fig. S4). In epithelial cells, TRMT112, SNU13, and NOP10 exhibited subtype-specific expression, with TRMT112 notably elevated in TNBC, consistent with its association with tumor aggressiveness (Supplementary Fig. S4A). Beyond tumor cells, RRMPs expression varied across immune subsets, including B cells, T cells, and macrophages (Supplementary Fig. S4B), suggesting a role in tumor-immune interactions.

### TRMT112 emerges as a key player in breast cancer progression and metastasis

**Fig. 3:**
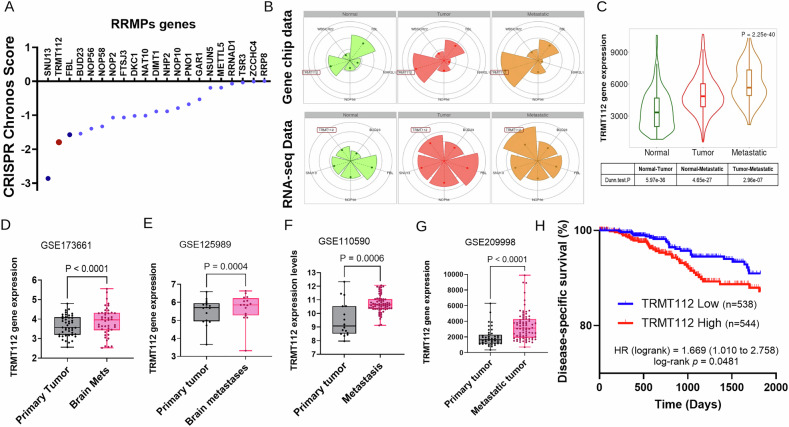
TRMT112 emerges as a key player in breast cancer progression and metastasis. **A** A scatter plot of CRISPR Chronos scores for RRMPs across 49 breast cancer cell lines shows SNU13, TRMT112 and FBL as top dependencies, indicating their essential role in cell survival (DepMap Public 22Q4+Score) (https://depmap.org/portal/), with more negative scores indicating a higher dependency. **B** Targetgram visualization of the top five hits from the Chronos CRISPR knockout screen (SNU13, TRMT112, FBL, BUD23, NOP56) reveals TRMT112 is uniquely upregulated in tumors and metastatic breast tissues, highlighting its role in tumor progression and metastasis. Data from gene chip and RNA-Seq were analyzed using the TNMplot tool. **C** A violin plot shows TRMT112 expression increases from normal to tumor and metastatic tissues, using TNMplot data from 33,520 samples (453 metastatic, 29,376 tumors, 3691 normal). This highlights TRMT112’s role in breast cancer progression and metastasis. **D** A boxplot shows a significantly higher TRMT112 expression in brain metastatic tumors (*n* = 45) and matched primary breast tumors (*n* = 45) using the GSE173661 dataset. **E** A boxplot displays TRMT112 expression levels are higher in brain metastases (*n* = 16) and matched primary breast cancers (*n* = 16) using data from the GSE125989 dataset. **F** A boxplot shows higher TRMT112 levels multiple metastases compared to matched primary tumors using the GSE110590 dataset. **G** A boxplot shows increased TRMT112 levels in metastatic tumors (*n* = 79) compared to primary breast tumors (*n* = 44) using data from the GSE209998 dataset. **H** Kaplan–Meier survival curve from TCGA-BRCA data indicates high TRMT112 expression (red) correlates with poorer five-year disease-specific survival (HR = 1.669, *p* = 0.0481), supporting its potential as a prognostic marker.

We queried a comprehensive CRISPR knockout screen across 49 breast cancer cell lines to evaluate the functional dependency of RRMPs. The CRISPR Chronos scores identified SNU13, TRMT112, and FBL as essential for cancer cell viability, as evidenced by their highly negative scores (Fig. 3A). To further investigate the expression dynamics of these top candidate RRMPs across different breast cancer contexts, we employed the Targetgram tool. This platform integrates gene chip and RNA-seq data to provide a detailed visualization of gene expression patterns across normal, tumor, and metastatic tissues, allowing for a refined understanding of their role in breast cancer progression. TRMT112 was significantly upregulated in tumor and metastatic tissues compared to normal, with pronounced expression in metastatic samples, underscoring its potential role in tumor progression and metastasis (Fig. 3B). Additional analysis using transcriptomic data from the NCBI-GEO integrated within TNMplot tool confirmed a progressive increase in TRMT112 expression from normal to mammary tumor to metastatic states (Fig. 3C).

Expression analysis, using data from the GSE173661 dataset in metastatic contexts, revealed a significant upregulation of TRMT112 in brain metastases compared to primary breast tumors, suggesting a role for TRMT112 in the propensity of breast cancer to metastasize to the brain (Fig. 3D). Confirmatory analysis using the GSE125989 dataset, which compared paired primary and brain metastatic samples, further supported this finding (Fig. 3E). Across multiple metastases from the same patients (GSE110590 dataset), TRMT112 expression was consistently elevated in metastatic tumors, reinforcing its role in metastatic progression (Fig. 3F). Broader analysis using the GSE209998 dataset from the AURORA US Metastasis Project corroborated these observations, with significantly greater TRMT112 expression in metastatic tumors (Fig. 3G). Disease-specific survival analysis from the TCGA-BRCA dataset highlighted that higher TRMT112 expression was significantly associated with poorer outcomes (Fig. 3H).

KMplot data analysis showed that TRMT112’s impact on survival varied across breast cancer subtypes, particularly affecting the basal subtype most adversely, thereby suggesting a subtype-specific role in breast cancer (Supplementary Fig. S5A). Protein level analysis using CPTAC data further indicated higher TRMT112 levels in TNBC compared to other subtypes (Supplementary Fig. S5B). Interestingly, while TRMT112 protein levels showed a potential beneficial trend in relapse-free survival among estrogen receptor-positive tumors, it demonstrated a detrimental association with overall survival in TNBC, reflecting its complex role across different breast cancer subtypes (Supplementary Fig. S5C, D).

### TRMT112 expression is increased in triple-negative breast cancer

**Fig. 4:**
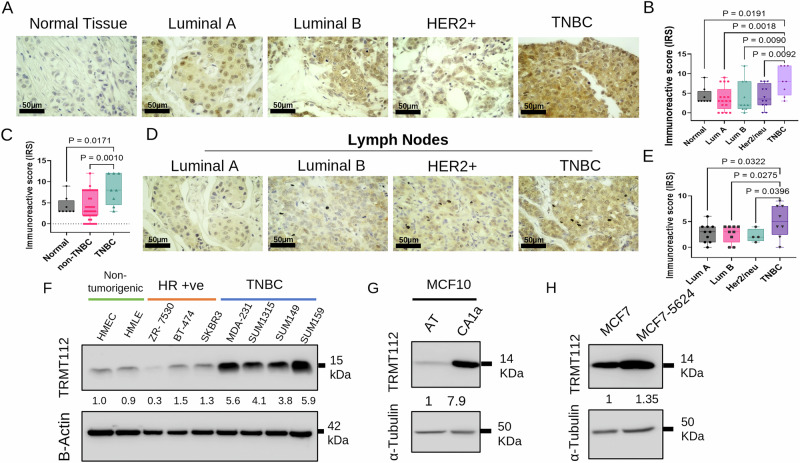
TRMT112 expression is high in TNBC primary tumors and lymph nodes. **A** Tissue microarray analysis visualized through immunohistochemistry, highlighting the expression of TRMT112 across various breast cancer subtypes and normal breast tissue. From left to right, the images display staining intensity in normal tissue, Luminal A, Luminal B, HER2+, and TNBC. Notably, TNBC shows significantly higher TRMT112 staining intensity compared to other subtypes. Scale bars = 50 µm. **B** A boxplot represents the immunoreactive scores (IRS), ranging from 0 to 12, for TRMT112 across various molecular subtypes of primary breast tumors, including Normal, Luminal A, Luminal B, HER2-enriched, and TNBC subtypes. **C** A boxplot illustrates IRS for TRMT112 Normal, non-TNBC, and TNBC categories. Notably, TNBC exhibits significantly higher IRS compared to other subtypes combined, highlighting its enhanced TRMT112 expression. **D** Immunohistochemical analysis of TRMT112 expression in lymph nodes from various breast cancer subtypes, Luminal A, Luminal B, HER2+, and TNBC. Representative images of lymph node metastases for each subtype are shown, with notably higher TRMT112 staining in TNBC tissues. Scale bars = 50 µm. **E** A boxplot shows the Immunoreactive Scores (IRS) of TRMT112 in lymph nodes across breast cancer subtypes: Luminal A, Luminal B, HER2-enriched, and TNBC. Notably, TNBC demonstrates significantly higher TRMT112 levels, suggesting its role in TNBC’s lymphatic spread and aggressiveness. **F** Western blot of TRMT112 expression in a range of breast cancer cell lines from non-tumorigenic (HMEC, HMLE) to hormone receptor-positive (ZR-7530, BT-474) and TNBC subtypes (MDA-MB-231, SUM1315, SUM149). β-Actin is used as a loading control. The numbers represent densitometric quantification of TRMT112 levels normalized to β-Actin. **G** Western blot for TRMT112 in MCF10AT (a premalignant cell line forming slow-growing tumor nodules) and its fully malignant and highly invasive counterpart, MCF10CA1a. TRMT112 expression is markedly higher in MCF10CA1a compared to the less invasive MCF10AT. α-Tubulin was used as a loading control. The numbers denote TRMT112 expression normalized to α-Tubulin. **H** Western blot shows TRMT112 levels in luminal subtype cell lines MCF7 and its more aggressive counterpart, MCF7-5624. MCF7-5624 displays increased TRMT112 expression compared to MCF7. α-Tubulin was used as a loading control; numbers indicate TRMT112 levels normalized to it.

We carried out a comprehensive immunohistochemical analysis of TRMT112 across various breast cancer subtypes to obtain additional-independent insight. We immunohistochemically assessed TRMT112 in 49 breast cancer tumors and observed distinct expression patterns, with the most pronounced staining in TNBC (Fig. 4A). Compared to normal breast tissue and other breast cancer subtypes TNBC exhibited significantly higher TRMT112 immunoreactivity, indicating an integral role of TRMT112 in the aggressiveness of this subtype (Fig. 4B, C). We further extended our analysis to matched lymph node tissues; once again TNBC demonstrated significantly elevated TRMT112 levels, underscoring its potential involvement in the metastatic spread characteristic of this aggressive cancer subtype (Fig. 4D, E).

We also analyzed TRMT112 expression across a spectrum of breast cancer cell lines, spanning non-tumorigenic to invasive TNBC subtypes. Relative to immortalized mammary epithelial HMEC cells or non-metastatic cells (HMLE, ZR-7530, BT-574, SKBR3), aggressive TNBC cells, particularly SUM159 and MDA-MB-231, express significantly higher levels of TRMT112 (Fig. 4F). Next, we contrasted TRMT112 expression from the tumorigenic non-metastatic cell line, MCF10AT with its isogenic, more aggressive, and metastatic counterpart, MCF10CA1a. This comparison revealed that TRMT112 protein expression is markedly higher in MCF10CA1a cells (Fig. 4G). This observation was consistent across another isogenic cell line pair; MCF7-5624, a more aggressive and metastatic derivative of the luminal MCF7 cells exhibited increased levels of TRMT112 compared to its parental line (Fig. 4H).

### TRMT112 regulates ribosome biogenesis, rRNA modification, and to a lesser extent, tRNA modification in TNBC cells

**Fig. 5:**
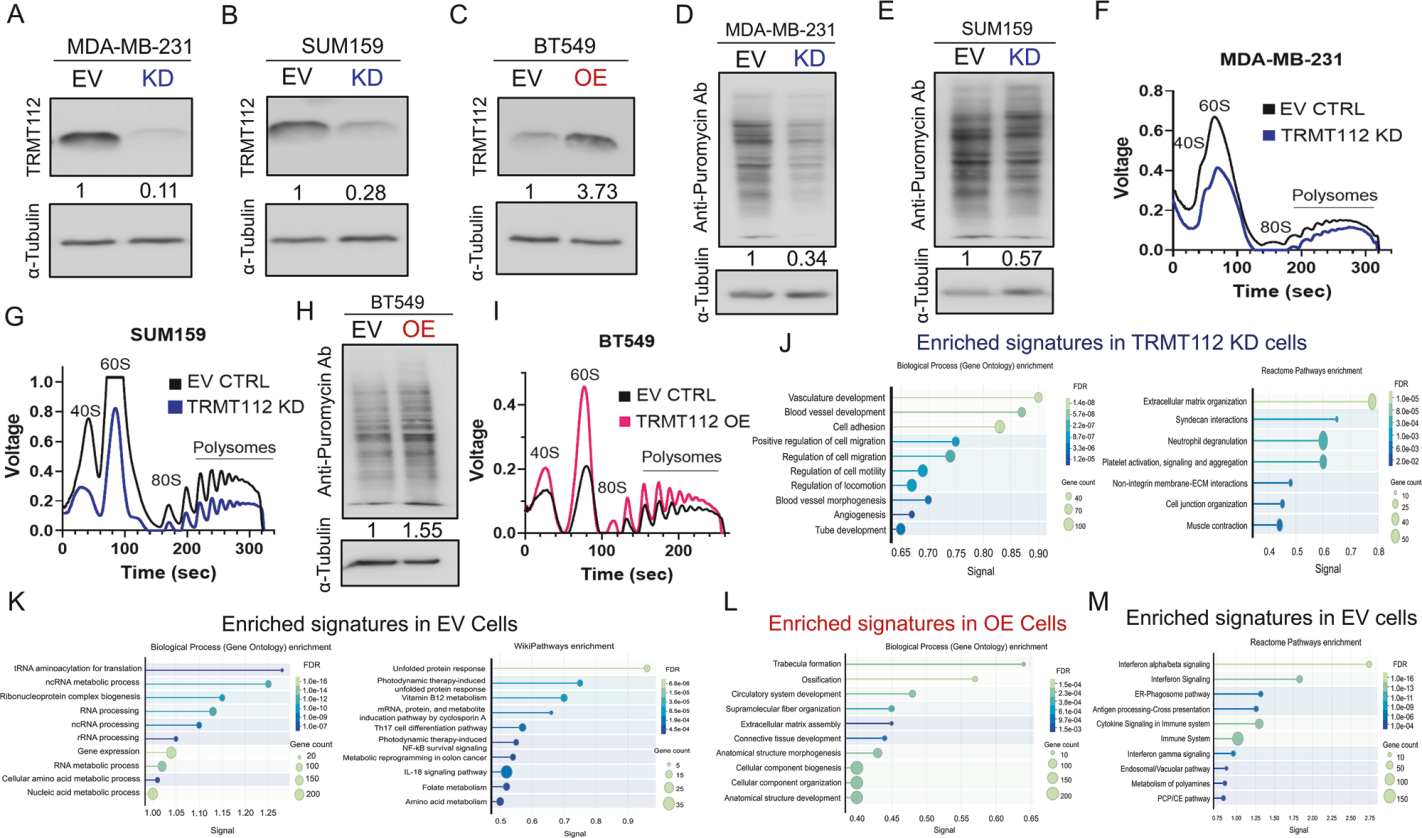
TRMT112 regulates protein synthesis and ribosome function in TNBC cells. Western blot analysis of TRMT112 in (**A**) MDA-MB-231 and (**B**) SUM159 cells, respectively, shows a significant reduction in TRMT112 levels in TRMT112 knockdown (KD) cells compared to empty vector (EV) control, with α-Tubulin as the loading control and for densitometric normalization. **C** Western blot analysis of TRMT112 in BT549 cells to confirm the TRMT112 overexpression in the TRMT112-overexpressing (OE) cells compared to empty vector control (EV) cells. α-Tubulin served as the loading control for densitometric analysis. Western blot analysis of puromycin incorporation into newly synthesized proteins in (**D**) MDA-MB-231 and (**E**) SUM159 cells, respectively. The comparison is between cells with EV and TRMT112 KD. The densitometric analysis of puromycin-labeled proteins in each lane was normalized to the corresponding α-Tubulin, which served as a loading control for quantification. Graphs represent polysome profiling of (**F**) MDA-MB-231 and (**G**) SUM159, respectively, illustrating ribosome distribution during translation. The profile reveals a marked decrease in the peaks representing the 40S and 60S ribosome subunits and the overall polysome population in TRMT112 KD cells compared to the control, underscoring the role of TRMT112 in maintaining efficient ribosome function and overall protein translation. **H** A Western blot analysis of puromycin incorporation into newly synthesized proteins in BT549 cells. It compares EV cells to TRMT112 OE cells. Puromycin-labeled protein levels were quantified by densitometry and normalized to α-Tubulin as a loading control. **I** A graph provides a detailed look at polysome profiling in BT549 OE cells compared to EV cells. The profiles demonstrate a marked increase in the peaks associated with the 40S and 60S ribosomal subunits and polysomes in the TRMT112 OE cells compared to EV cells. This elevation indicates an increase in ribosomal assembly and function, aligning with the increased protein synthesis observed in the puromycin incorporation assay. **J** STRING-db functional enrichment analysis of all actively translated mRNAs in TRMT112 KD cells (MDA-MB-231 and SUM159) shows an enrichment of genes involved in extracellular matrix organization, adhesion, and differentiation. **K** Functional enrichment analysis of all differentially translated genes in EV cells (MDA-MB-231 and SUM159) reveals significant enrichment in ribosome biogenesis, translational regulation, metabolic processes, and pathways linked to tumor growth and proliferation. **L** STRING-db functional analysis shows that TRMT112 overexpression in BT549 enhances translation of genes involved in ossification, and collagen formation, processes linked to increased matrix stiffening and metastatic potential. **M** STRING-db analysis reveals that BT549 EV cells are enriched in immune-related pathways, interferon signaling, antigen processing, and cytokine signaling, indicative of an anti-tumor immune response. These findings suggest that TRMT112 overexpression shifts the translational program towards a more invasive and metastatic phenotype while suppressing immune-related processes.

Data thus far shows that TRMT112 is upregulated in TNBC and further enriched in metastases. Given TRMT112’s reported role in rRNA and tRNA modifications, we next investigated whether modulation of TRMT112 alters the different steps of ribosome biogenesis. First, we silenced TRMT112 in SUM159, and MDA-MB-231 cells (Fig. 5A, B), and overexpressed TRMT112 in BT549 (Fig. 5C). TRMT112 knockdown (KD) in MDA-MB-231 and SUM159 cells showed a significant reduction in RNA Pol I activity, as evidenced by decreased transcription of 5′ ETS regions (Supplementary Fig. S6A, B), whereas TRMT112 overexpression (OE) in BT549 cells showed a pronounced increase in RNA Pol I activity (Supplementary Fig. S6C). Extending this analysis to rRNA maturation and processing, quantitative PCR across the 45S precursor revealed broad processing defects in TRMT112 KD cells. Conversely, TRMT112 OE led to a marked accumulation of processing intermediates, indicating increased rRNA processing (Supplementary Fig. S6D–G).

Given TRMT112’s established interactions with methyltransferases, we next examined rRNA and tRNA modifications. Readthrough assays demonstrated that TRMT112 KD increased readthrough at the 18S m6A1832 and m7G1639 sites, consistent with reduced methylation (Supplementary Fig. S7A–C), whereas TRMT112 OE reduced read-through mainly at the m7G1639 site (Supplementary Fig. S7D). TRMT112 KD had little to no effect on wobble uridine (mcm⁵U34) modification in tRNA-Lys(TTT) in SUM159, and MDA-MB-231 cells, while TRMT112 OE reduced readthrough in BT549 cells (Supplementary Fig. S7E–H). These results support a dominant role for TRMT112 in 18S rRNA methylation, with only a limited effect on tRNA modification in TNBC cells.

To explore whether these site-specific modification changes reflect differential regulation of TRMT112 cofactors, we analyzed the TCGA-BRCA CPTAC cohort. TRMT112 protein levels showed strong positive correlations with METTL5, THUMPD2, TRMT11 and ALKBH8, but not with BUD23 or THUMPD3 (Supplementary Fig. S8A). Consistent with these correlations, silencing TRMT112 in SUM159 and MDA-MB-231 cells sharply reduced METTL5, THUMPD2, ALKBH8 and TRMT11 transcripts, whereas TRMT112 OE produced the reciprocal increase of these cofactors (Supplementary Fig. S8B–D). Together, the data show that TRMT112 maintains the expression of specific methyltransferases, and that the magnitude of their downregulation determines the severity and specificity of downstream RNA-modification defects.

### TRMT112 regulates protein synthesis and ribosome activity in TNBC cells

To assess the impact of TRMT112-dependent rRNA modifications on translation, we performed polysome profiling and puromycin incorporation assays. TRMT112 KD in MDA-MB-231 and SUM159 cells led to a significant decrease in protein synthesis, as evidenced by reduced puromycin incorporation (Fig. 5D, E) and altered polysome profiles with diminished 40S, 60S, 80S, and polysome peaks (Fig. 5F, G). In contrast, TRMT112 OE enhanced protein synthesis, indicated by increased puromycin incorporation (Fig. 5H) and elevated ribosome profiling peaks (Fig. 5I).

Immunoblotting revealed that TRMT112 KD reduced levels of the translation initiation factor eIF4A1 and the phosphorylated eIF2α and 4E-BP1 in SUM159 cells (Supplementary Fig. S9A), further supporting impaired translation initiation upon TRMT112 loss. Conversely, OE increased eIF4A1 abundance in BT549 cells, while phosphorylation of 4EBP-1 was elevated relative to controls (Supplementary Fig. S9B), demonstrating that TRMT112 upregulation strengthens both ribosome activity and translation initiation. Consistent with these observations, analysis of the CPTAC-BRCA cohort revealed strong positive correlations between TRMT112 protein levels and several key translation factors, including eEF1A1, eIF4A1, eIF2A, 4E-BP1, and eIF4E (Supplementary Fig. S9C), supporting the clinical relevance of these regulatory interactions.

To further elucidate the impact of TRMT112 on translational regulation, we performed RNA sequencing on polysome-associated mRNAs from TRMT112 KD compared to empty vector (EV) cells (Supplementary Fig. S10A). TRMT112 KD resulted in an overall decrease in polysomes and a distinct translational program (Supplementary Fig. S10B–D), characterized by upregulation of genes involved in adhesion, differentiation, and cytoskeletal remodeling, including ATP1B1, LMCD1, TPM1, and MDK, consistent with a shift toward a less invasive phenotype (Supplementary Fig. S11A). Analysis of the TCGA-BRCA CPTAC dataset showed positive correlations between TRMT112 and genes actively translated in EV cells, while genes preferentially translated in KD cells exhibited negative correlations (Supplementary Fig. S11B, C). Gene-set analysis confirmed suppression of pro-tumorigenic pathways with enrichment of adhesion-related processes in TRMT112 KD cells compared to EV cells (Fig. 5J, K).

Reciprocally, polysome-RNA-seq in TRMT112 OE BT549 cells revealed a shift toward mRNAs involved in stromal remodeling and collagen formation, the hallmarks of metastatic progression (Fig. 5L, Supplementary Fig. S12A, B). Conversely, immune-related pathways, including antigen processing and interferon signaling, were suppressed in TRMT112 OE cells (Fig. 5M, Supplementary Fig. S12C), suggesting that TRMT112 promotes tumor aggressiveness by enhancing pro-metastatic translational programs while dampening anti-tumor immunity. Together, these findings position TRMT112 as a central regulator of translational control in TNBC, acting through both ribosome activity and translation factor modulation to promote aggressive tumor phenotypes by enhancing pro-metastatic and immune-evasive translational programs.

### TRMT112 promotes tumorigenic behavior and metastatic potential in TNBC cells

**Fig. 6:**
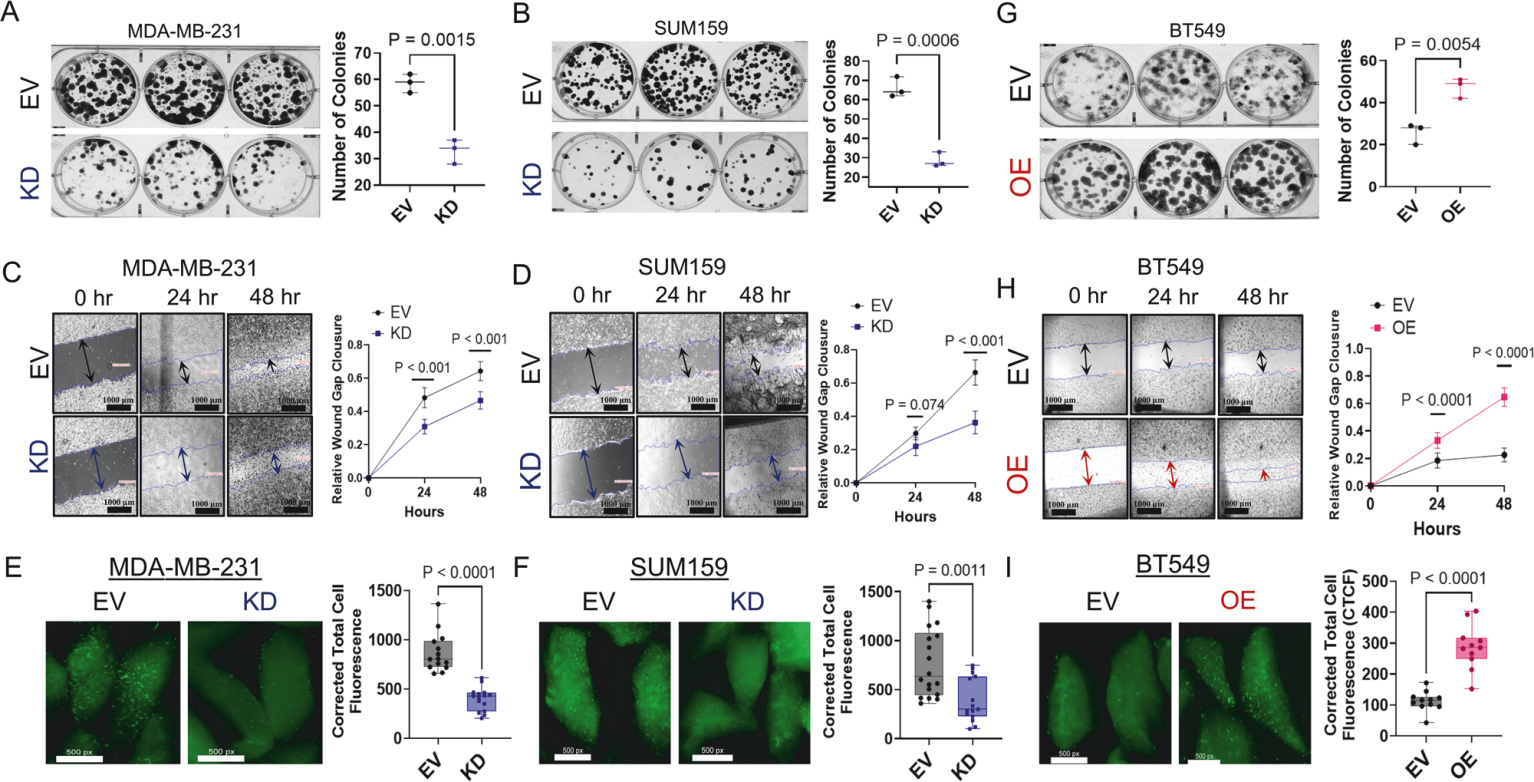
TRMT112 modulates proliferation, migration, and metastatic potential in TNBC cells. Panels display results from a colony formation assay in MDA-MB-231 (**A**) and SUM159 (**B**) cells. The assay compares EV cells with TRMT112 KD cells. After 7 days of incubation, the KD cells exhibit a significant reduction in the number of colonies formed, indicating that TRMT112 plays a crucial role in cell proliferation and colony stability. Representative images of wound healing assay in MDA-MB-231 (**C**) and SUM159 (**D**) cells that measures the migration capability of cells. In TRMT112 KD cells, there is a significant delay in wound closure compared to control cells. The graphs quantitatively show that the distance traveled by KD cells is substantially less, suggesting that TRMT112 is important for cellular motility. A panel assesses metastatic and seeding potential using an ex vivo pulmonary seeding assay in MDA-MB-231 (**E**) and SUM159 (**F**) cells. Fluorescence imaging of lung sections from TRMT112 KD cells shows significantly reduced seeding efficiency compared to control cells, highlighting the impact of TRMT112 on the metastatic behavior of breast cancer cells. Scale bars = 500 px. **G** Colony formation assay comparing EV BT549 cells with TRMT112 OE cells. The results show a significant increase in the number of colonies formed by the overexpressing cells, indicating that TRMT112 enhances colony stability and cell proliferation capabilities. **H** A wound healing assay evaluates the migration capabilities of the BT549 cells. TRMT112 OE cells demonstrate accelerated wound closure compared to controls within 24 and 48 h. This suggests that TRMT112 plays a vital role in promoting cellular motility. **I** In an ex vivo pulmonary seeding assay, fluorescence imaging quantifies the metastatic potential of the cells. TRMT112 OE cells show a marked increase in seeding efficiency within lung tissues, as indicated by the significantly higher corrected total cell fluorescence (CTCF), highlighting TRMT112’s influence on enhancing metastatic behavior. Scale bars = 500 px.

Next, we sought to determine how TRMT112 modulates key tumorigenic and invasive features. Despite comparable rates of proliferation between the TRMT112 KD and EV cells (Supplementary Fig S13A, B), TRMT112 KD in MDA-MB-231 and SUM159 cells resulted in a significant reduction in colony formation (Fig. 6A, B). Wound healing assays further demonstrated impaired motility, with delayed wound closure and decreased migration distance in TRMT112-depleted TNBC cells (Fig. 6C, D, Supplementary Fig. S13C, D). Consistently, transwell assays revealed a marked reduction in migration following TRMT112 KD (Supplementary Fig. S13E), and Matrigel invasion assays confirmed that TRMT112 depletion significantly diminished invasive capacity (Supplementary Fig. S13F).

Further morphological analyses through three-dimensional culture assays revealed that TRMT112 KD cells assumed less invasive structures, characterized by decreased protrusive features and increased spherical structures (Supplementary Fig. S14A–D). This morphological change is suggestive of diminished invasive capacity. The pulmonary metastasis assay (PuMA) corroborated these findings, where TRMT112 KD resulted in significantly reduced seeding efficiency in lung sections, emphasizing the protein’s role in exacerbating metastatic traits (Fig. 6E, F, Supplementary Fig. S14E).

While TRMT112 OE in BT549 cells did not significantly impact their proliferation (Supplementary Fig. S15A), it enhanced tumorigenic properties, notably foci formation and suggestive of a robust capacity to induce density-independent growth (Fig. 6G). Additionally, the TRMT112-OE cells exhibited increased motility in wound healing assays (Fig. 6H, Supplementary Fig. S15B). Consistently, transwell migration assays revealed a significantly higher number of migrating cells following TRMT112 OE compared with EV controls (Supplementary Fig. S15C), and Matrigel invasion assays confirmed that TRMT112 OE cells exhibited markedly elevated invasive capacity (Supplementary Fig. S15D). Moreover, in three-dimensional culture, OE cells formed aggressive, invasive structures with pronounced protrusions, highlighting their enhanced invasive potential (Supplementary Fig. S15E, F). Furthermore, PuMA demonstrated that TRMT112 OE significantly increased the metastatic seeding capability within lung tissues, marked by elevated metastatic lesions (Fig. 6I).

**Fig. 7:**
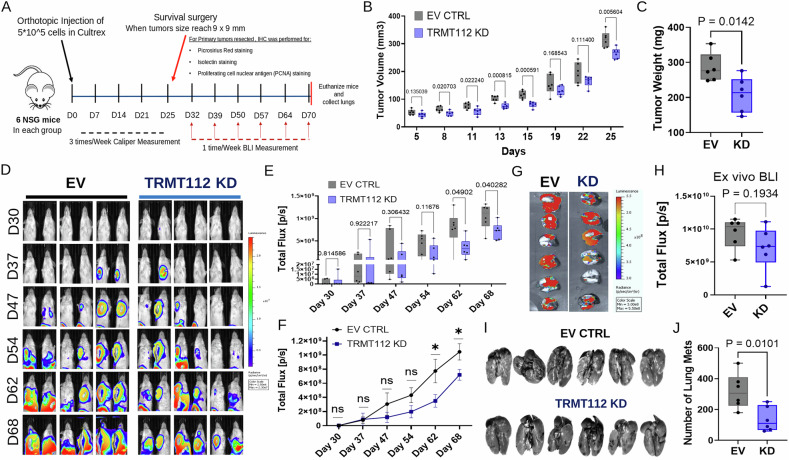
TRMT112 knockdown suppresses primary tumor growth and metastatic progression in an orthotopic breast cancer model. **A** Schematic overview of the experimental timeline. Orthotopic injections of breast cancer cells (MDA-MB-231 - 5 × 10⁵) in a Cultrex matrix were performed on D0. Tumor size was measured three times per week using calipers. Upon reaching 9 × 9 mm, survival surgery was conducted for excision of primary tumors, followed by bioluminescence imaging once per week to monitor metastatic progression. **B** Tumor volume measurements at the indicated time points in mice injected with EV or TRMT112 KD cells. Data represent mean ± SD of measurements from 6 mice per group. Statistical significance was determined using a two-tailed Student’s *t* test; *p*-values are shown for each time point. **C** Box plot representing the weight of primary tumors excised from EV and TRMT112 KD groups. Tumor weight was significantly reduced in the TRMT112 KD group compared to EV CTRL. Statistical analysis was performed using a two-tailed Student’s *t* test (*p* = 0.0142). **D** Bioluminescence imaging (BLI) showing metastatic progression at various time points (Day 30–Day 68). Representative images demonstrate reduced metastatic spread in TRMT112 KD mice compared to EV group. **E** Box plot representation of total flux at each time point. While no significant differences were observed at early stages (D30–D47), TRMT112 KD mice exhibited significantly lower metastatic burden from D54 onward. p-values for each comparison are indicated. **F** Longitudinal analysis of total bioluminescent flux (p/s) from D30–D68 reveals a significant reduction in metastatic progression in TRMT112 KD mice compared to EV group, particularly at later time points. Statistical significance was assessed using a two-tailed Student’s *t* test (*p < 0.05; ns not significant). **G** Representative ex vivo BLI images of lungs from EV and TRMT112 KD mice following a 10-s exposure illustrate metastatic burden. **H** Quantification of total ex vivo bioluminescent flux shows a trend toward reduced metastatic seeding in TRMT112 KD lungs. **I** Representative images of lung metastases harvested at endpoint from mice in the EV and TRMT112 KD groups. TRMT112 knockdown significantly reduces metastatic burden in the lungs. **J** Box plot showing the number of lung metastases in EV CTRL and TRMT112 KD groups at the endpoint (D70). TRMT112 knockdown significantly reduced the number of lung metastases. Statistical analysis was performed using a two-tailed Student’s *t* test (*p* = 0.0101).

To evaluate the impact of TRMT112 on tumor progression, we orthotopically injected NSG mice with MDA-MB-231 EV CTRL or KD cells (Fig. 7A). Tumor growth was monitored using caliper measurements three times per week. TRMT112 KD tumors exhibited a significant reduction in both tumor diameter and volume compared to the control group throughout the study duration (Fig. 7B, Supplementary Fig. S16A, B). On day 25, survival surgery was performed, and primary tumors were resected. Tumors from the TRMT112 KD group were significantly smaller, with a marked reduction in weight compared to the EV CTRL group (Fig. 7C, Supplementary Fig. S16C).

To assess metastatic dissemination, bioluminescence imaging (BLI) was performed weekly following primary tumor excision. Longitudinal BLI analysis revealed a significant reduction in metastatic burden in TRMT112 KD mice, particularly at later time points (Days 54–68) (Fig. 7D–F). At the experimental endpoint, ex vivo BLI analysis of lung tissues showed a trend toward reduced metastatic seeding in TRMT112 KD mice (Fig. 7G, H). Representative images of excised lungs demonstrated a notable reduction in metastatic burden in TRMT112 KD mice compared to controls. Quantification of metastatic nodules confirmed a significant decrease in the number of lung metastases in the TRMT112 KD group (Fig. 7I, J). Collectively, these findings highlight the key role of TRMT112 in promoting tumor progression and metastatic dissemination in breast cancer.

## Discussion

Our findings assign a novel role for RRMPs beyond their traditionally understood functions in ribosomal biogenesis and define a key role for RRMPs in cancer development, progression, and potential therapeutic targets, particularly in breast cancer. The variability in RRMPs expression suggests that these proteins could serve as critical nodes in cancer-specific regulatory networks [5, 7, 8]. Our findings demonstrate that RRMPs are highly expressed in aggressive breast cancer subtypes, particularly TNBC, highlighting the pivotal role of rRNA modifications in supporting tumor growth and promoting metastatic processes [10, 11]. This is supported by our in vitro and ex vivo findings, where modulation of a specific RRMP, TRMT112, markedly influenced cell proliferation, migration, invasion, and metastatic potential.

These results corroborate with other studies suggesting that alterations in ribosomal machinery components can drive tumorigenesis through changes in protein synthesis and cellular stress responses [13, 16]. The significant association between elevated RRMPs expression and poor survival outcomes as revealed in our analyses aligns with emerging literature that links dysregulated protein synthesis machinery to cancer aggressiveness and resistance to therapy [28]. Furthermore, alterations in RRMPs correlate with genomic instability markers, suggesting their role in promoting tumor heterogeneity and aggressive cancer traits [9, 25, 29].

The TRMT112 protein, a highly conserved methyltransferase activator, plays a central role in ribosome biogenesis by stabilizing rRNA methyltransferases such as WBSCR22/BUD23 and METTL5, which mediate 18S rRNA modifications critical for ribosome assembly and translation fidelity. In addition, TRMT112 forms complexes with several tRNA methyltransferases, including TRMT11, ALKBH8, and THUMPD2/3, thereby extending its function beyond rRNA processing to the regulation of tRNA modification and translational control [13, 14, 19, 22]. Furthermore, its synergy with its partners, especially METTL5, drives tumorigenesis by enhancing ribosome assembly and the translation of mRNAs involved in fatty acid metabolism [15, 30].

Our RNA-seq analysis of polysome fractions revealed that TRMT112 reshapes the translational landscape, particularly affecting mRNAs encoding proteins involved in adhesion, cytoskeletal remodeling, and invasion. This is consistent with the emerging view that ribosome composition and rRNA modifications are not merely housekeeping processes but can actively reprogram translation to favor tumor-promoting pathways and dampen anti-tumor immunity [25, 31–33].

Our in vitro and in vivo findings underscore TRMT112 as a potential therapeutic vulnerability in TNBC. As TRMT112 is indispensable for the stability and activity of multiple methyltransferases [13, 16, 17, 19], its inhibition could disrupt mainly rRNA modification, modestly tRNA modification, and translational control simultaneously in TNBC, thereby exerting pleiotropic anti-tumor effects. Targeting TRMT112 or its critical interactions may represent a novel strategy to suppress ribosome-driven oncogenic programs, particularly in TNBC, where therapeutic options remain limited [34, 35].

In conclusion, our study demonstrates that RRMPs, and particularly TRMT112, are key regulators of ribosome biogenesis, RNA modification, and translational control in breast cancer. By linking rRNA modifications to protein synthesis, tumor cell invasion, and immune evasion, TRMT112 emerges as a multifunctional regulator of cancer progression. These findings highlight TRMT112 as both a prognostic biomarker and a promising therapeutic target, providing a mechanistic foundation for interventions aimed at disrupting TRMT112-driven translational programs in aggressive breast cancers, especially in TNBC.

## Material and methods

Detailed materials and methods are provided in Supplemental Materials and Methods and Key Resources Table.

### Statistical analysis

All statistical analyses were performed using GraphPad Prism 10 and R software. Data normalization and transformations were applied as needed, including log2 transformation for gene expression data and z-scores for enrichment analyses. For comparative gene expression across conditions and breast cancer subtypes, statistical tests such as Student’s *t* tests, Mann–Whitney U tests, and one-way ANOVA were utilized based on the data characteristics. Comparative analyses, including Kaplan–Meier survival curves, were used to assess RRMPs expression’s impact on patient outcomes, with hazard ratios calculated using the log-rank test. Multiple testing adjustments, such as the Bonferroni correction, were applied where needed to control for Type I errors. Additionally, differential expression of RRMPs in breast cancer was visualized using ggplot2 in R, with dot size representing statistical significance (-log10(*p*-value)) and color reflecting fold-change.

## Supplementary information

Supplemental materials and Methods

Supplemental Figures and Legends

Related Manuscript File

Related Manuscript File

Related Manuscript File

## Data Availability

Publicly available datasets analyzed in this study include The Cancer Genome Atlas (TCGA) breast cancer cohort and Molecular Taxonomy of Breast Cancer International Consortium (METABRIC) data, both accessed through the cBioPortal for Cancer Genomics (https://www.cbioportal.org/). Additional datasets used for expression and survival analyses include GSE110590, GSE57968, and GSE209998 (AURORA US Metastasis Project), retrieved from the NCBI Gene Expression Omnibus (GEO). Single-cell RNA-sequencing data were obtained from GSE176078 and analyzed via the Broad Institute Single Cell Portal. Protein expression data from the TCGA-BRCA CPTAC cohort were downloaded from cBioPortal, and CRISPR Chronos dependency scores were obtained from the DepMap 22Q4 Public release (https://depmap.org/portal/). The raw and processed RNA-seq data generated from the polysome fractionation experiments have been deposited in the NCBI Gene Expression Omnibus (GEO) under accession number GSE311075. All other data supporting the findings of this study are available within the article and its Supplementary Information files.
