# Supplementary material for: TRMT112 drives a tumor growth and metastasis-promoting program in triple-negative breast cancer
Source: Cell Death Differ. 2026 Jan 8;33(6):1192–202. doi: 10.1038/s41418-025-01643-z (PMC13246786; doi:10.1038/s41418-025-01643-z)
Supplement: Supplementary file 1 — Supplemental materials and Methods [file 41418_2025_1643_MOESM1_ESM.pdf]

Supplemental Materials and Methods

TRMT112 Drives A Tumor Growth and Metastasis-Promoting Program in Triple-Negative Breast Cancer

Amr R. Elhamamsy[1], Brandon J. Metge[1], Mohamed H. Elbahoty[1], Bhavyasree Papineni[1], Heba Allah M. Alsheikh[1], Dongquan Chen[2,3], Rajeev S. Samant[1,3], and Lalita A. Shevde[1,3†]

[1]Department of Pathology, University of Alabama at Birmingham, AL, USA

[2]Division of General Internal Medicine and Population Science, Department of Medicine, University of Alabama at Birmingham, Birmingham, AL, USA

[3]O'Neal Comprehensive Cancer Center, University of Alabama at Birmingham, AL, USA

[†]Corresponding author

Address correspondence to: Lalita A. Shevde, WTI 320D, 1824 6th Avenue South, Birmingham, AL 35233. Email: lsamant@uab.edu; Phone: 205-975-6261

Genomic Data Retrieval

In selecting RRMPs for our analysis, we conducted an extensive review of the literature and database searches, selecting genes known or predicted to be involved in rRNA modification. Our selection included genes associated with various rRNA modification mechanisms, categorized broadly based on their functional involvement. These categories included H/ACA box snoRNPs (e.g., dyskerin, GAR1, NHP2, NOP10) involved in pseudouridylation, C/D box snoRNPs (e.g., fibrillarin, NOP56, NOP58, SNU13) responsible for 2'-O-methylation, and other essential methyltransferases and decay factors. This systematic classification of RRMPs based on their specific roles in rRNA processing enabled a more targeted investigation into how alterations in these proteins might impact ribosomal function and contribute to oncogenic processes in various cancers.

For our study on the role of RRMPs in cancer, we primarily utilized genomic and clinical data from The Cancer Genome Atlas (TCGA) and the Molecular Taxonomy of Breast Cancer International Consortium (METABRIC). TCGA offered comprehensive whole-genome sequencing, RNA-Seq, and clinical outcome data across various cancer types, while METABRIC provided detailed genomic, transcriptomic, and clinical data specifically for breast cancer. These resources allowed for a broad analysis of RRMPs alterations and their implications across multiple cancer types and subtypes. Additional datasets such as GSE110590, GSE57968, and GSE209998 from the AURORA US Metastasis Project were also integrated to enrich our analyses, particularly focusing on metastatic breast cancer.

Genomic alteration data for RRMPs were retrieved from cBioPortal (https://www.cbioportal.org/) using TCGA datasets. Three alteration types—structural variants, mutations, and copy number alterations (CNAs)—were analyzed across multiple cancer types. Frequencies were calculated and visualized using GraphPad Prism, with color coding to represent each alteration type. Heatmaps of RRMPs alterations (amplifications, deletions, and mutations) across cancer types were generated using the Morpheus tool, based on TCGA data. Genes were categorized by their rRNA modification roles (H/ACA box, C/D box, and methyltransferases), and color intensity reflected alteration frequency.

To assess genomic instability in breast cancer, boxplots comparing Tumor Mutational Burden (TMB), Aneuploidy Score, and Fraction of Genome Altered between samples with and without RRMPs alterations were created using GraphPad Prism. Statistical significance was determined using the Wilcoxon test. RRMPs alteration frequencies in Ductal and Lobular Carcinomas were further stratified by mRNA expression levels, with bar graphs illustrating higher alteration rates in tumors with elevated RRMPs expression. These analyses provided insights into the potential role of RRMPs in cancer progression and genomic instability.

Bioinformatics Analyses

In our study, the bioinformatics analyses utilized specific tools optimized for handling and visualizing complex genomic data sets. For Principal Component Analysis, we utilized GEPIA2 (Gene Expression Profiling Interactive Analysis), an enhanced web server that facilitates robust functional annotation of gene expression data. GEPIA2 was

particularly chosen for its user-friendly interface and its ability to integrate and analyze RNA sequencing expression data from the TCGA and GTEx projects simultaneously. This tool enabled us to perform PCA, providing a clear visual distinction of expression levels across various samples. This analysis was crucial for identifying patterns of RRMPs expression among different cancer types and normal tissues, helping to delineate the distinct genetic landscapes that characterize various forms of cancer.

For the visualization of RRMPs expression across different cancer types and the construction of gene expression similarity matrices, we employed the Morpheus tool available from the Broad Institute. Morpheus is versatile for its matrix visualization and analysis capabilities, allowing for the generation of heatmaps and the computation of hierarchical clustering and similarity matrices. By inputting normalized expression data into Morpheus, we were able to create heatmaps that display the differential expression of RRMPs, and similarity matrices that reveal the correlation patterns among RRMPs across cancerous and normal samples. These visualizations provided a detailed portrayal of the expression dynamics and the potential cooperative functions or disruptions among RRMPs in various cancer contexts, enriching our understanding of their roles in oncogenesis and progression.

Single-cell RNA sequencing (scRNA-seq) data from GSE176078 was analyzed to assess the expression patterns of rRNA modification-associated genes across diverse cell populations in human breast cancer. Processed data were visualized using the Single Cell Portal (Broad Institute), where scaled mean expression and the percentage of expressing cells were quantified. Differential expression analysis was performed

across normal epithelial, tumor epithelial (HER2+ and TNBC), immune, and stromal cell compartments.

Protein expression data from the TCGA-BRCA CPTAC cohort (n = 104) were obtained through cBioPortal. Protein z-scores for TRMT112 and its known cofactors (BUD23, METTL5, THUMPD2, THUMPD3, TRMT11, and ALKBH8), key translation factors (eEF1A1, eIF4A1, eIF2A, 4E-BP1, and eIF4E), and selected TRMT112-dependent translational targets (ASNS, VRK1, ATP1B1, LMCD1, TPM1, and MDK) were extracted. Correlation analyses were performed in GraphPad Prism 10 using Pearson correlation coefficients, and statistical significance was determined by two-tailed p-values.

Single-Sample Gene Set Enrichment Analysis (ssGSEA)

The ssGSEA was conducted using the GenePattern platform, a comprehensive suite for genomic analysis. This analysis involved mapping the expression data of our defined RRMPs gene set against reference gene sets to calculate enrichment scores for each sample. This process allowed us to quantify how closely the expression of RRMPs in individual cancer samples correlated with predefined reference sets, indicative of their biological activity. Normalization methods were applied to the gene expression data prior to analysis to minimize technical variability and ensure robust comparative insights across the study cohort.

Survival Analysis

For survival analysis, we employed a combination of tools to generate and analyze Kaplan-Meier survival curves. The initial threshold for high and low RRMPs expression

was determined using the Xena Browser, which allowed us to set cutoff thresholds based on the distribution of RRMPs expression levels across the patient population. Groups were then stratified accordingly, and survival curves were generated using GraphPad Prism. Hazard ratios (HRs) were calculated within GraphPad, and the statistical significance of differences between groups was assessed using the log-rank test. Multiple testing corrections, such as the Bonferroni method, were applied where necessary to control for false discovery rates.

Additionally, we utilized KMplot (https://kmplot.com/analysis/) to further explore survival outcomes based on both gene and protein expression data. This tool allowed us to generate survival curves using publicly available datasets and provided an additional layer of validation for the prognostic significance of RRMPs. Kaplan-Meier plots from KMplot, based on either mRNA or protein expression levels, were incorporated into the analysis to corroborate findings from the publicly available datasets. Together, these analyses offered a robust framework for determining the prognostic impact of RRMPs in breast cancer and other cancer types.

Generation of CRISPR Chronos Scores Scatter Plot

CRISPR Chronos scores were obtained from the Dependency Map (DepMap) Public 22Q4 release via the DepMap Portal (https://depmap.org/portal/). These scores indicate the essentiality of RRMPs in breast cancer cell lines, with more negative values suggesting higher cellular dependency. We selected the mean Chronos scores for each RRMPs across 49 breast cancer cell lines to assess their critical roles in cell survival. A scatter plot was created to visualize these dependencies, highlighting genes like

TRMT112 and SNU13 as vital for breast cancer cell viability. This analysis was performed using Python's Matplotlib library to ensure precise and clear graphical representation.

Targetgram Visualization Using TNMplot Tool

Expression patterns of top RRMP genes identified from the CRISPR screen—SNU13, TRMT112, FBL, BUD23, and NOP56—were analyzed across normal, tumor, and metastatic breast tissues using the TNMplot tool. This web-based platform allows for comparative analysis of gene expression data from gene chip and RNA-Seq technologies. A Targetgram was generated to delineate the expression variations of these genes, particularly noting the pronounced expression of TRMT112 in tumor and metastatic contexts. The visualization aimed to underscore the gene's involvement in tumor progression and its potential as a therapeutic target. The TNMplot's graphical outputs provided a comprehensive overview, facilitating the identification of RRMPs with significant roles in the pathology of breast cancer at different stages.

Cell Culture

Breast cancer cell lines used in this study were maintained under specific culture conditions to ensure optimal growth and experimental consistency. SUM159 cells were cultured in DMEM/F-12 medium (ThermoFisher Scientific, Cat# 11330032) supplemented with 5% heat-inactivated fetal bovine serum (FBS) (ThermoFisher Scientific, Cat# A5256701), 5 µg/mL insulin (Millipore Sigma, Cat# I5500-500MG), and 1 µg/mL hydrocortisone (Millipore Sigma, Cat# H0888-1G). BT-549 cells were

maintained in RPMI 1640 medium (ThermoFisher Scientific, Cat# 22400105) supplemented with 10% heat-inactivated FBS, while MDA-MB-231 cells were cultured in DMEM/F-12 medium supplemented with 5% heat-inactivated FBS. MCF10A and MCF10CA1a cl.1 cells were grown in DMEM/F-12 medium supplemented with 5% horse serum (ThermoFisher Scientific), 10 µg/mL insulin (Millipore Sigma), 25 ng/mL human epidermal growth factor (hEGF; Millipore Sigma), 250 ng/mL hydrocortisone (Millipore Sigma), and 100 ng/mL cholera toxin (Millipore Sigma). SKBr3 cells were cultured in McCoy's 5A medium (ThermoFisher Scientific) with 10% FBS. SUM149 cells were maintained in DMEM/F-12 medium containing 5% FBS, 5 µg/mL insulin (Millipore Sigma), and 1 µg/mL hydrocortisone (Millipore Sigma). HMEC and HMLE cells were cultured in DMEM/F-12 medium supplemented with 10 ng/mL EGF (Millipore Sigma), 500 ng/mL hydrocortisone (Millipore Sigma), and 10 µg/mL insulin (Millipore Sigma). BT-474 cells were maintained in RPMI-1640 medium with 10% FBS, 1% L-glutamine, and 10 µg/mL insulin (Millipore Sigma). SUM1315 cells were grown in DMEM/F-12 medium supplemented with 5% FBS, 500 µL insulin (from a 10 mg/mL stock solution), and 500 µL EGF (from a 25 µg/mL stock solution). ZR-7530 cells were cultured in RPMI-1640 medium with 10% FBS and 1% sodium pyruvate, whereas MCF-7 and MCF7-5624 cells were maintained in DMEM/F-12 medium supplemented with 10% FBS and 10 µg/mL insulin (Millipore Sigma). All culture media were devoid of antibiotics and antimycotics. Cells were maintained at 37°C in a humidified atmosphere containing 5% CO2. Routine passaging was performed using trypsinization, and cells were monitored regularly for mycoplasma contamination. These standardized conditions ensured optimal growth and minimized the risk of contamination.

For TRMT112 knockdown (KD), GIPZ TRMT112 Lentiviral shRNA (Horizon Discovery, Cat# RHS4531-EG51504) was transfected into cells using Lipofectamine 2000 (Thermo Fisher) according to the manufacturer's protocol. GIPZ Non-silencing Lentiviral shRNA (Cat# RHS4346) was used as a control under the same conditions. For TRMT112 overexpression (OE), we used the ORF expression clone for TRMT112 (GeneCopoeia, Cat# EX-J0039-M02-B), with the pReceiver-M02 empty vector as the control (Cat# EX-NEG-M02-B). Transfection was carried out using Lipofectamine 2000, and selection of transfected cells was performed using G418 Sulfate (500 µg/mL, Thermo Fisher, Cat# 10131027) for overexpression and Puromycin (0.5 µg/mL, Thermo Fisher, Cat# 227420100) for knockdown constructs.

All culture media were devoid of antibiotics and antimycotics. Cells were maintained at 37°C in a humidified atmosphere containing 5% CO_2. Routine passaging was performed using trypsinization, and cells were monitored regularly for mycoplasma contamination. These standardized conditions ensured optimal growth and minimized the risk of contamination.

Cell Proliferation Assays

To evaluate the effect of TRMT112 modulation on breast cancer cell proliferation, MDA-MB-231, SUM159, and BT549 cell lines were cultured under two different media conditions: complete media and 1% FBS. Cells were plated in twelve-well plates at a density of 25,000 cells per well. MDA-MB-231 and SUM159 cells were cultured in DMEM/F-12 media supplemented with 5% FBS, while BT549 cells were grown in RPMI-1640 media with 10% FBS. For nutrient limiting conditions, the media was

supplemented with only 1% FBS. Cell counts were recorded over a 5-day period. Growth rates were compared between EV CTRL, TRMT112 KD for MDA-MB-231 and SUM159, and TRMT112 OE for BT549 cells.

Puromycin Incorporation and Ribosome Profiling

Puromycin incorporation assays were conducted in MDA-MB-231, SUM159, and BT549 cell lines. After reaching 60-70% confluence, puromycin was added to the media at a final concentration of 10 µg/mL for 30 minutes at 37°C. Cells were then lysed, and whole-cell lysates were subjected to western blot analysis to detect puromycin-labeled nascent proteins using anti-puromycin antibody, clone 12D10 (Sigma-Aldrich # MABE343), and using α-tubulin as a loading control. Densitometric analysis was performed to quantify the levels of puromycin incorporation, which was normalized to the control.

For ribosome profiling, cells were grown to 60-70% confluence in 10 cm dishes and treated with cycloheximide (100 µg/mL) for 5 minutes at 37°C to arrest ribosomes on mRNA. Cells were washed twice with ice-cold PBS containing cycloheximide and lysed in polysome lysis buffer (20 mM Tris-HCl, 10 mM MgCl2, 300 mM NaCl, 1% Triton X-100, 100 µg/mL cycloheximide, 0.1 U/µL RNase inhibitor, 0.5 mg/mL heparin, and 1x Halt protease inhibitors). Lysates were clarified by centrifugation at 13,000 rpm for 10 minutes at 4°C. The protein concentration was measured, and 2.5 mg of total protein was loaded onto pre-prepared 10-50% sucrose gradients. Gradients were ultracentrifuged at 35,000 rpm for 3 hours 20 minutes at 4°C using a Beckman SW41Ti rotor. Following ultracentrifugation, gradients were fractionated using the Brandel SYN-

202 Density Gradient Fractionation System. This system facilitates the precise collection of fractions by displacing the gradient from the bottom up with a dense chase solution, while simultaneously providing a continuous absorbance profile using a UA-6 detector with 254 and 280 nm filters, and a R1 fraction collector.

Western Blotting

Cell lysates were prepared by lysing cells directly in 2X Laemmli buffer containing β-mercaptoethanol, followed by heating at 95°C for five minutes. Proteins were separated via SDS-PAGE and transferred onto polyvinylidene difluoride (PVDF) membranes. Membranes were blocked in 5% non-fat dry milk in TBST (Tris-buffered saline with 0.1% Tween-20) for one hour at room temperature and then incubated overnight at 4°C with primary antibodies.

The following primary antibodies were used: anti-TRMT112 (1:1000, Sigma-Aldrich, Cat# HPA040006), anti-Puromycin (1:5000, Sigma-Aldrich, Cat# MABE343), and anti-eIF4A1 (1:1000, Cell Signaling Technologies, Cat# 2490), anti-phospho-eIF2α (1:1000, Cell Signaling Technologies, Cat# 3398, anti-eIF2α (1:1000, Cell Signaling Technologies, Cat#2103), anti-4EBP-1 (1:1000, Cell Signaling Technologies, Cat# 9644), and anti-phospho-4EBP-1 (1:1000, Cell Signaling Technologies, Cat# 9451). Anti-α-tubulin (1:1000, Cell Signaling Technologies, Cat# 3873S) or anti-β-actin (1:50,000, Sigma-Aldrich, Cat# A3854) were used as loading controls.

After washing with TBST, membranes were incubated with HRP-conjugated secondary antibodies (anti-rabbit, GE Healthcare, Cat# NA934; anti-mouse, GE Healthcare, Cat# NA931) for one hour at room temperature. Blots were developed using Pierce™ ECL

Western Blotting Substrate (Thermo Scientific, Cat# 32209) or ECL™ Prime Western Blotting System (Cytiva, Cat # RPN2232). Blots were then imaged with an Amersham Imager 600. Densitometric analysis was performed using Image Studio Lite (LI-COR Biosciences) software, with relative protein expression normalized to α-tubulin or β-actin, as indicated.

Analysis of RNA Polymerase I transcriptional activity

RNA was extracted using the RNeasy Mini Kit (Qiagen) according to the manufacturer's instructions. To assess RNA Polymerase I (Pol I) activity, the abundance of transient 5′ external transcribed spacer (5′ETS) rRNA was quantified by real-time PCR. One microgram of total RNA was reverse transcribed into cDNA using the High-Capacity cDNA Reverse Transcription Kit (Thermo Fisher Scientific). Quantitative PCR was carried out with 1:100 diluted cDNA using Maxima SYBR Green Master Mix (Thermo Fisher Scientific) on an Applied Biosystems Step One Plus Real-Time PCR system.
Two primer sets were used to amplify the 5′ETS region as reported previously [1]: forward 5′-GAACGGTGGTGTGTCGTT-3′ and reverse 5′-GCGTCTCGTCTCGTCTCACT-3′ (851–961), and forward 5′-CAGGTGTTTCCTCGTACCG-3′ and reverse 5′-GCTACCATAACGGAGGCAGA-3′ (1297–1483). β-actin served as the internal reference control, using forward 5′-CATGTACGTTGCTATCCAGGC-3′ and reverse 5′-CTCCTTAATGTCACGCACGAT-3′ primers.

Relative changes in 5′ETS transcript abundance were calculated using the ΔΔCT method. Fold-change values were normalized to β-actin and expressed relative to

empty vector (EV) controls, thereby enabling quantitative evaluation of Pol I transcriptional activity under TRMT112 modulation.

Analysis of ribosomal RNA processing

Total RNA was extracted from TRMT112 knockdown (KD) and overexpression (OE) cell lines using the RNeasy Mini Kit (Qiagen), following the manufacturer's protocol. One microgram of RNA was reverse transcribed into cDNA using the High-Capacity cDNA Reverse Transcription Kit (Thermo Fisher Scientific). Quantitative PCR was performed with 1:100 diluted cDNA using Maxima SYBR Green Master Mix (Thermo Fisher Scientific), with β-actin mRNA serving as the internal control for normalization.

Precursor rRNA transcripts were analyzed using primer sets described previously [2]. These included primers targeting the 45S (forward: 5′-GAACGGTGGTGTGTCGTT-3′; reverse: 5′-GCGTCTCGTCTCGTCTCACT-3′), the 18S 5′ junction (forward: 5′-GCCGCGCTCTACCTTACCTACCT-3′; reverse: 5′-CAGACATGCATGGCTTAATCTTTG-3′), the 18S 3′ junction (forward: 5′-AGTCGTAACAAGGTTTCCGTAGGT-3′; reverse: 5′-CCTCCGGGCTCCGTTAAT-3′), the 5.8S 5′ junction (forward: 5′-TACGACTCTTAGCGGTGGATCA-3′; reverse: 5′-TCACATTAATTCTCGCAGCTAGCT-3′), the 5.8S 3′ junction (forward: 5′-GAATTGCAGGACACATTGATCATC-3′; reverse: 5′-GGCAAGCGACGCTCAGA-3′), and the 28S 5′ junction (forward: 5′-CCGAGACGCGACCTCAGAT-3′; reverse: 5′-TCCGCTGACTAATATGCTTAAATTCA-3′).

To quantify mature rRNAs, primers were designed against the 18S (forward: 5′-GATGGTAGTCGCCGTGCC-3′; reverse: 5′-GCCTGCTGCCTTCCTTGG-3′), the 5.8S

(forward: 5′-ACTCGGCTCGTGCGTC-3′; reverse: 5′-GCGACGCTCAGACAGG-3′), and the 28S (forward: 5′-GTGACGCGCATGAATGGA-3′; reverse: 5′-TGTGGTTTCGCTGGATAGTAGGT-3′) transcripts. For internal normalization, β-actin primers were used (forward: 5′-CATGTACGTTGCTATCCAGGC-3′; reverse: 5′-CTCCTTAATGTCACGCACGAT-3′).

Relative transcript abundance was calculated as fold change normalized to β-actin, and values were expressed relative to empty vector (EV) controls. This analysis enabled us to assess the impact of TRMT112 modulation on rRNA transcription and processing.

Quantitative real-time PCR analysis of TRMT112 interactors

Total RNA was extracted from TRMT112 knockdown (KD) and overexpression (OE) cell lines using the RNeasy Mini Kit (Qiagen), according to the manufacturer’s instructions. One microgram of RNA was reverse transcribed into cDNA using the High-Capacity cDNA Reverse Transcription Kit (Thermo Fisher Scientific). Quantitative PCR was then performed using 1:10 diluted cDNA and Maxima SYBR Green Master Mix (Thermo Fisher Scientific) on an Applied Biosystems Step One Plus Real-Time PCR system. β-actin was used as the internal reference for normalization.

Primer sequences were obtained from the PrimerBank database [3]. For β-actin, the primers were forward 5′-CATGTACGTTGCTATCCAGGC-3′ and reverse 5′-CTCCTTAATGTCACGCACGAT-3′ (ID: 4501885a1). For TRMT112, the primers were forward 5′-GGTCCGTATCTGCCCTGTG-3′ and reverse 5′-GGATCAGACGCAAGTTATCGG-3′ (ID: 7705476c1). For BUD23, the primers were forward 5′-CCCTGTTACCTGCTGGATATTG-3′ and reverse 5′-

ATGCAACCATCAAATGTGCCT-3′ (ID: 356874773c2). METTL5 was amplified using forward 5′-AAGGAACTAGAGAGTCGCCTG-3′ and reverse 5′-GCGGCCTGGTAGGATACTG-3′ (ID: 92859574c1). For THUMPD3, the primers were forward 5′-CCAACTCCTAGATGTGAACCTTC-3′ and reverse 5′-AGTGGCTCCAATAGTGACTAGAA-3′ (ID: 166197707c1). For THUMPD2, the primers were forward 5′-CAGCAGAGCTTACATCAAGACA-3′ and reverse 5′-GTAACTGTGAGTCGCTGACATC-3′ (ID: 254553430c1). For ALKBH8, the primers were forward 5′-ATGGACAGCAACCATCAAAGTAA-3′ and reverse 5′-GGCTCTGAGTGGCATAGGATAC-3′ (ID: 195927055c1). For TRMT11, the primers were forward 5′-TCCGCCTGCCGGAAATAAAG-3′ and reverse 5′-ACACACTGTCCGTTTCATCAAAT-3′ (ID: 94420682c1).
Relative transcript abundance was determined using the ΔΔCT method. Fold-change values were normalized to β-actin, and data were expressed relative to empty vector (EV) controls. This experimental design enabled quantitative evaluation of how TRMT112 modulation affects the expression of its key cofactors and interacting partners.

Low-dNTP/Mn^{2+} RT-stop qPCR and relative read-through

Total RNA was extracted in TRIzol (Thermo Fisher; Cat # 15596018), and the <200-nt fraction was purified using the Zymo Research RNA Clean & Concentrator kit (Zymo research; Cat # R1017) with the small-RNA workflow, following the manufacturer's instructions. Purified small RNAs were quantified with the Qubit RNA HS assay (Thermo Fisher). Reverse-transcriptase pausing at modified sites was assayed by paired RT

reactions per sample: a control RT containing 0.5 mM total dNTP and no Mn^{2+}, and a low-stringency RT containing 2 µM total dNTP plus 0.5 mM $MnCl_2$ (Sigma Aldrich; Cat # 221279). Induro Reverse Transcriptase (NEB; Cat # M0681) was used with the supplied 5× buffer and 0.1 M DTT; RNase inhibitor (Thermo Fisher; Cat # AM2696) was present in all reactions. Typical 20 µL RTs contained RNA (≤250 ng), 1 µL gene-specific primer (5 µM; the same reverse primer used for qPCR), 4 µL buffer, 2 µL DTT, 1 µL inhibitor, 0.5 µL Induro RT, and water to volume. Reactions were denatured at 65 °C for 5 min, chilled on ice for 2 min, incubated at 25 °C for 2 min and 42 °C overnight (~14 h), and heat-inactivated at 95 °C for 1 min. cDNA was diluted 1:30 and quantified by SYBR Green qPCR (Applied Biosystems; Cat # A25742) (20 µL: 10 µL 2× mix, 200 nM each primer, 4 µL cDNA; 95 °C 2 min; 40 cycles of 95 °C 15 s, 60 °C 30 s), followed by melt-curve analysis. No-RT and no-template controls were included. All primers sequences are provided in the Key Resources Table.

Within a sample, RT-stall was summarized as ΔCt_stall = Ct_low-dNTP − Ct_control, where larger positive values indicate stronger pausing (higher modification occupancy). For the relative read-through metric shown in the figure and table, Cq values from the low-dNTP RT only were first normalized to the housekeeping transcript measured in the same RNA (β-actin for rRNA assays or U6 for tRNA assays) to obtain dCt_low = Ct_low – CT_housekeeping. Condition effects were then referenced to the appropriate control sample (e.g., EV): ΔΔCt = dCt_low (KD/OE) − dCt_low (CTRL), and relative read-through = 2^(−ΔΔCt). Thus, values >1 denote more read-through (weaker pausing/hypomodification) compared with the reference, whereas values <1 denote less read-through (stronger pausing/higher modification).

RNA-seq Analysis of Actively Translated Transcripts

Fractions corresponding to actively translating polysomes were pooled, and total RNA was extracted using the RNeasy Mini Kit (Qiagen) following the manufacturer's protocol. RNA integrity was assessed using an Agilent Bioanalyzer 2100. RNA-seq libraries were generated from purified polysomal RNA using the TruSeq Stranded Total RNA Library Prep Kit (Illumina) after rRNA depletion. Libraries were sequenced on the Illumina NovaSeq 6000 platform, generating paired-end 150-bp reads. Raw sequencing data in FASTQ format underwent quality control and adapter trimming using Trimmomatic. High-quality reads were aligned to the human genome (GRCh38) using HISAT2, and PCR duplicates were removed with SAMtools. Read counts were normalized as Reads Per Kilobase per Million mapped reads (RPKM), and differential translation analysis was performed using DESeq2 in the Partek Genomics Suite. To identify biological pathways enriched among differentially translated transcripts, gene ontology (GO) and pathway enrichment analyses were conducted using STRING-db (https://string-db.org/).

Colony Formation Assay

Cells were plated at low density, with 100–300 cells per well, depending on the cell line, in six-well plates containing complete growth medium. Plates were incubated under standard culture conditions (37°C, 5% CO_2) for 7–10 days, allowing colonies to form. Culture medium was replaced every three days to maintain optimal growth conditions. At the experimental endpoint, media was removed, and wells were gently washed with phosphate-buffered saline (PBS) to remove residual debris. Cells were then fixed with

3.7% formaldehyde (equivalent to 10% formalin) for 15 minutes at room temperature. Following fixation, formaldehyde was aspirated, and cells were stained with 0.1% crystal violet for 15 minutes to visualize colonies. Excess stain was carefully removed by rinsing wells with water for 15–30 minutes. Plates were air-dried at room temperature before imaging and quantification.

3D Culture Protocol

Cells were cultured using Cultrex 3D Culture Matrix Reduced Growth Factor Basement Membrane (Fisher, Cat# 3445-010-01). Cultrex was thawed overnight at 4°C and handled on ice to prevent solidification. Pre-chilled pipet tips were used for Cultrex manipulation. A total of 150 µl of Cultrex was added to each well of an eight-well glass chamber slide (Millipore, Cat# PEZGS0816), spread evenly, and incubated at 37°C for at least 30 minutes to allow solidification. Cells were detached, counted, and resuspended in complete growth media at 10,000 cells/ml, then mixed 1:1 with 2X Assay media (4% Cultrex). A total of 500 µl of this mixture (5,000 cells per well) was added to each well, and the chamber slides were immediately incubated at 37°C. 3D cultures were maintained until structures formed (5-21 days), with media changes every other day using fresh media containing 2% Cultrex. Once the 3D structures were fully formed, cells were stained using NucBlue Live Cell Stain ReadyProbes and incubated for 20-25 minutes. Imaging was performed using a Nikon microscope at 10X magnification, capturing DAPI and bright-field images.

Wound Healing Assay

Cells were seeded at a density of 250,000 cells per well in a 6-well plate and incubated overnight to allow for confluence. The following day, the medium was replaced with fresh media containing 1% FBS to limit cell proliferation and emphasize migration. A scratch was made in the cell monolayer using a 20 µL pipette tip to generate a uniform wound. Imaging of the wound was performed immediately after the scratch (0 hours), and at 24- and 48-hours post-scratch using a Nikon Eclipse Ti-U microscope at 10X and 20X magnification. The Nikon NIS-Elements AR software was used to capture images and quantify the wound area and distance traveled by the cells.

Invasion assay

The invasive capacity of TRMT112 knockdown (KD) and overexpression (OE) cells was evaluated using BD BioCoat Matrigel invasion chambers (Corning, 8.0 µm pore size, Cat. No. 354480). Inserts were equilibrated at room temperature, rehydrated for 2 hours with 500 µl of serum-free medium at 37 °C, and the medium was then carefully removed. A total of 20,000 cells, suspended in 500 µl of serum-free medium, were seeded into each insert. The lower wells were filled with 750 µl of serum-free medium supplemented with 10 µg/ml fibronectin as the chemoattractant. Cells were incubated at 37 °C for 12 hours, after which the inserts were washed and fixed in 4% paraformaldehyde for 10 minutes, followed by staining with 0.5% crystal violet (Difco Laboratories) for 15 minutes. Filters were rinsed with water and air-dried. For each condition, experiments were performed in triplicate, and four representative fields per insert were imaged using a Nikon Eclipse E200LED microscope at 10× magnification. The number of invaded cells per field was quantified using ImageJ software [4].

Migration assay

Cell migration was assessed using BD Falcon cell culture inserts (8.0 µm pore size, Cat. No. 353097). Inserts were pre-coated overnight at 4 °C with 600 µl of serum-free medium containing 6 ng/ml gelatin, followed by rehydration with 150 µl of serum-free medium at room temperature for 90 minutes. After removal of the medium, inserts were transferred to wells containing 750 µl of serum-supplemented growth medium. TRMT112 KD and OE cells (50,000 per insert) were suspended in 500 µl of serum-free medium and seeded into the upper chambers. Cells were incubated at 37 °C for 4 hours, after which non-migrated cells were removed, and migrated cells were fixed in 4% paraformaldehyde for 10 minutes and stained with 0.5% crystal violet for 15 minutes. Filters were rinsed in water and air-dried. Experiments were performed in triplicate, and four images per insert were captured using a Nikon Eclipse E200LED microscope at 10× magnification. Migrated cells were quantified using ImageJ software.

Immunohistochemical Analysis

Tissue microarrays (TMAs) were used to assess TRMT112 expression across breast cancer subtypes, including matched metastatic and normal breast tissues. The BRM961a microarray panel (TissueArray.com) consisted of 96 formalin-fixed, paraffin-embedded (FFPE) tissue cores (1.5 mm diameter, 5 µm thick) from 48 cases, encompassing 48 breast carcinomas, 35 matched metastatic tissues, and 12 adjacent normal tissues. Slides were stored at 4°C and baked for 2 hours at 60°C prior to staining to maintain antigen integrity and prevent tissue detachment during processing.

Deparaffinization was carried out by immersing slides sequentially in xylene and graded ethanol (100%, 95%, and 70%). Antigen retrieval was performed in sodium citrate buffer (10 mM, pH 6.0) by heating in a microwave for 5 minutes. After cooling, the slides were rinsed in distilled water and outlined with a hydrophobic PapPen to confine staining to the tissue sections. Blocking was performed with Dual Endogenous Enzyme Block (Dako EnVision+ Dual Link System-HRP) for 15 minutes at room temperature. Slides were incubated overnight at 4°C with a primary anti-TRMT112 antibody (1:500 dilution, HPA040006, Sigma-Aldrich). After washing, sections were incubated with anti-rabbit HRP-labeled polymer for 40 minutes, and staining was visualized with DAB+ substrate for 7 minutes. Slides were counterstained with Harris Hematoxylin, dehydrated in graded ethanol, and cleared in xylene. Stained sections were scanned using a Leica Aperio ScanScope CS at 40X magnification. Image analysis was performed using Aperio ImageScope software (version 12.3.3), assessing staining intensity and the percentage of positive cells to calculate immunoreactive scores (IRS). Subcellular localization of TRMT112 was categorized as cytoplasmic, nuclear, or mixed across breast cancer subtypes, including Luminal A, Luminal B, HER2-enriched, and TNBC.

Ex Vivo Pulmonary Metastasis Assay (PuMA)

A total of 2 × 10^5 cells were injected intravenously into female mice. Fifteen minutes post-injection, the mice were euthanized, and the trachea was cannulated and perfused with a 1:1 mixture of 1.2% low-melting agarose and Culture Medium 1. Lungs were excised, cooled in PBS at 4°C for 20 minutes, and sectioned into 1–2 mm thick slices.

Lung sections were cultured on Gelfoam sponges pre-soaked in Culture Medium 2 at 37°C in 5% CO_2, with media changes every other day and regular flipping of lung sections. Culture Medium 1 consisted of M-199 medium supplemented with 2.0 µg/mL insulin, 0.2 µg/mL hydrocortisone, 0.2 µg/mL retinyl acetate, 200 U/mL penicillin, 200 µg/mL streptomycin, and 7.5% sodium bicarbonate. Culture Medium 2 contained M-199 medium with 1.0 µg/mL insulin, 0.1 µg/mL hydrocortisone, 0.1 µg/mL retinyl acetate, 100 U/mL penicillin, 100 µg/mL streptomycin, and 7.5% sodium bicarbonate. Fluorescent imaging was conducted every two to three days using a utilizing a Nikon SMZ800 stereo zoom microscope, and subsequent image analysis was performed using ImageJ software. Metastatic burden was quantified using the corrected total cell fluorescence (CTCF) of areas of tumor cells across lung sections. All procedures were approved by the Institutional Animal Care and Use Committee (IACUC) and adhered to NIH guidelines.

Orthotopic Breast Cancer Model and Tumor Growth Monitoring

To assess the impact of TRMT112 KD on tumor growth and metastasis, an orthotopic breast cancer model was employed using NOD/SCID gamma (NSG) mice. A total of 5×10^5 MDA-MB-231 cells expressing either an empty vector (EV CTRL) or TRMT112 KD were suspended in Cultrex (Basement Membrane Extract, Type 3, Bio-Techne, cat # 3632-010-02) and orthotopically injected into the mammary fat pad of 6-week-old female NSG mice (n = 6 per group). Tumor growth was monitored three times per week using digital caliper measurements, and tumor volume was calculated using the formula: $V = (\text{length} \times \text{width}^2)/2$. Maximal tumor diameter permitted by UAB ethics

committee and our IACUC protocol is 10 mm. Upon reaching a tumor size of 9 × 9 mm, mice underwent survival surgery for tumor excision, and primary tumors were processed for histological and immunohistochemical analyses.

To evaluate metastatic dissemination, longitudinal bioluminescence imaging (BLI) was performed on days 30, 37, 47, 54, 62, and 68 post-injection using the IVIS Lumina III (PerkinElmer). Imaging, data acquisition and analysis were performed using Living Image software (PerkinElmer). Bioluminescent signals, quantified as total photon flux (p/s), were acquired for each mouse, with comparisons made between EV CTRL and TRMT112 KD groups. Statistical significance at each time point was assessed using a two-tailed Student's t-test. At the experimental endpoint (D70), mice were euthanized, and lungs were harvested to evaluate metastatic burden. Ex vivo BLI was performed on lung tissues using the IVIS Lumina III, with a 10-second exposure to capture luciferase-expressing metastatic lesions. Quantification of total flux (p/s) was conducted using Living Image software, and statistical differences between groups were determined using a two-tailed Student's t-test. Primary tumors were excised, weighed, and processed for histological and immunohistochemical staining. Lung metastases were also quantified macroscopically.

Use of Large Language Model

During the preparation of this work the authors used GPT-4 to improve the readability of the manuscript. After using this tool/service, the authors reviewed and edited the content as needed and take full responsibility for the content of the publication.

References

1. Peltonen K, Colis L, Liu H, Trivedi R, Moubarek MS, Moore HM, et al. A targeting modality for destruction of RNA polymerase I that possesses anticancer activity. Cancer Cell. 2014;25(1):77-90.
2. Kwon I, Xiang S, Kato M, Wu L, Theodoropoulos P, Wang T, et al. Poly-dipeptides encoded by the C9orf72 repeats bind nucleoli, impede RNA biogenesis, and kill cells. Science. 2014;345(6201):1139-45.
3. Spandidos A, Wang X, Wang H, Seed B. PrimerBank: a resource of human and mouse PCR primer pairs for gene expression detection and quantification. Nucleic Acids Res. 2010;38(Database issue):D792-9.
4. Schneider CA, Rasband WS, Eliceiri KW. NIH Image to ImageJ: 25 years of image analysis. Nat Methods. 2012;9(7):671-5.
