# Supplementary material for: TRMT112 drives a tumor growth and metastasis-promoting program in triple-negative breast cancer
Source: Cell Death Differ. 2026 Jan 8;33(6):1192–202. doi: 10.1038/s41418-025-01643-z (PMC13246786; doi:10.1038/s41418-025-01643-z)
Supplement: Supplementary file 2 — Supplemental Figures and Legends [file 41418_2025_1643_MOESM2_ESM.pdf]

Supplementary Data

TRMT112 Drives A Tumor Growth and Metastasis-Promoting Program in Triple-Negative Breast Cancer

Running title: *TRMT112 and Cancer Progression*

Amr R. Elhamamsy[1], Brandon J. Metge[1], Mohamed H. Elbahoty[1], Bhavyasree Papineni[1], Heba Allah M. Alsheikh[1], Dongquan Chen[2,3], Rajeev S. Samant[1,3], and Lalita A. Shevde[1,3†]

[1]Department of Pathology, University of Alabama at Birmingham, AL, USA

[2]Division of General Internal Medicine and Population Science, Department of Medicine, University of Alabama at Birmingham, Birmingham, AL, USA

[3]O'Neal Comprehensive Cancer Center, University of Alabama at Birmingham, AL, USA

[†]Corresponding author

Address correspondence to: Lalita A. Shevde, WTI 320D, 1824 6th Avenue South, Birmingham, AL 35233. Email: lsamant@uab.edu; Phone: 205-975-6261

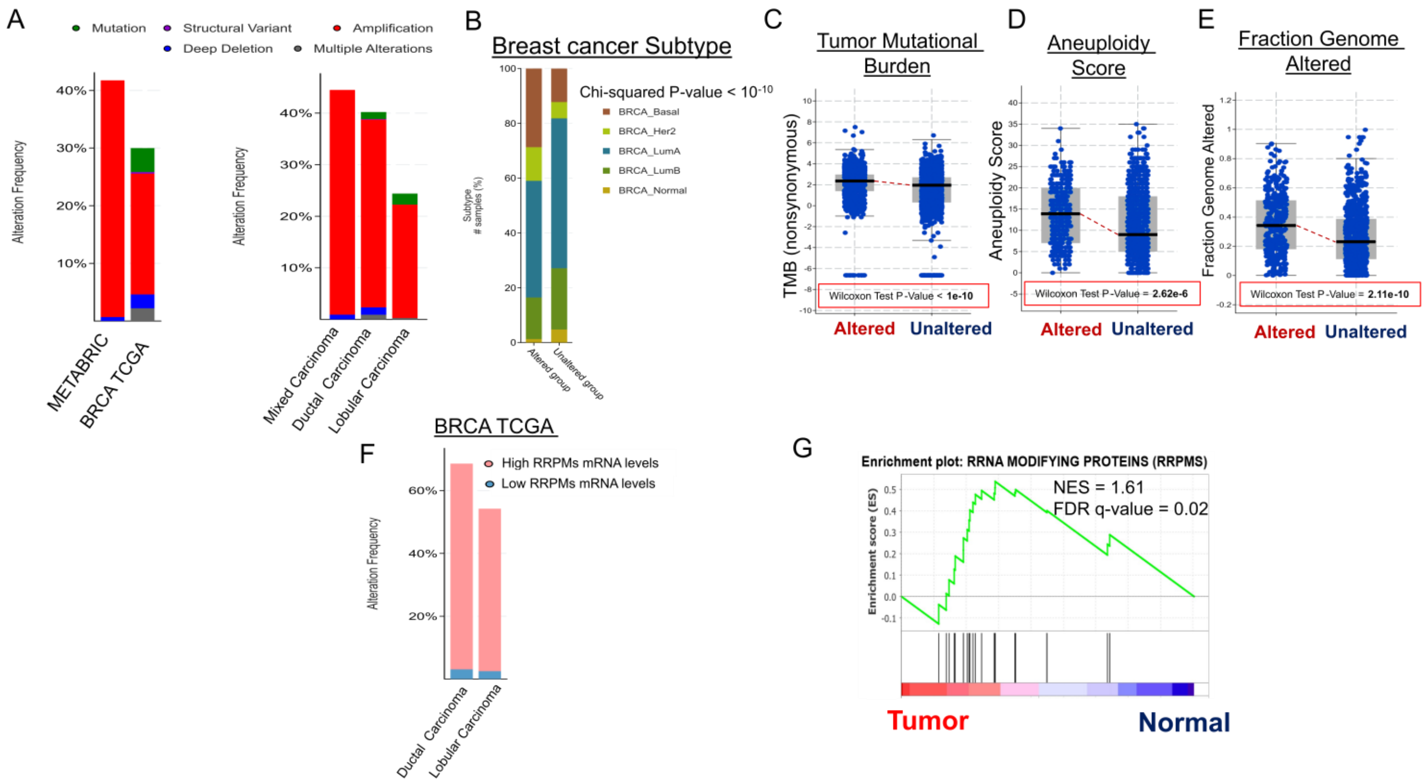

Supp Figure 1. RRMPs Alterations Are Strongly Associated with Genomic Instability and Specific Breast Cancer Subtypes

(A) Bar graph showing the frequencies of genetic alterations in Ribosomal RNA Modifying Proteins (RRMPs) across breast cancer subtypes using METABRIC and TCGA BRCA data. Five alteration types are displayed: Mutations (green), Structural Variants (purple), Amplifications (red), Deep Deletions (blue), and Multiple Alterations (gray). The left panel compares datasets, while the right shows alterations by subtype (Mixed, Ductal, Lobular). Notably, RRMPs gene amplifications are prevalent, underscoring their oncogenic potential and therapeutic relevance.

(B) Bar graph illustrating the distribution of breast cancer subtypes by RRMPs alteration status. Basal subtype shows a significantly higher prevalence of RRMPs alterations

(chi-squared p < 10^-10), highlighting a strong association between these alterations and aggressive subtypes.

(C-E) Boxplots from TCGA BRCA data reveal genomic instability metrics. (C) Tumor Mutational Burden (TMB) is significantly higher in RRMPs-altered (structurally mutated) tumors (Wilcoxon p < 1e-10). (D) Aneuploidy Scores are elevated in altered cases (Wilcoxon p = 2.62e-6), and (E) Fraction of Genome Altered is greater in the same group (Wilcoxon p = 2.11e-10), linking RRMPs alterations to increased genomic instability.

(F) Bar graph comparing RRMPs alteration frequency in Ductal and Lobular Carcinomas stratified by RRMPs mRNA expression. Tumors with high RRMPs expression, particularly Ductal Carcinomas, show increased alteration frequencies, suggesting a connection between elevated expression and genomic changes.

(G) Gene Set Enrichment Analysis (GSEA) enrichment plot demonstrates significant RRMPs signature enrichment in tumor versus normal tissues (NES = 1.61, FDR q = 0.02), supporting the role of RRMPs in tumorigenesis.

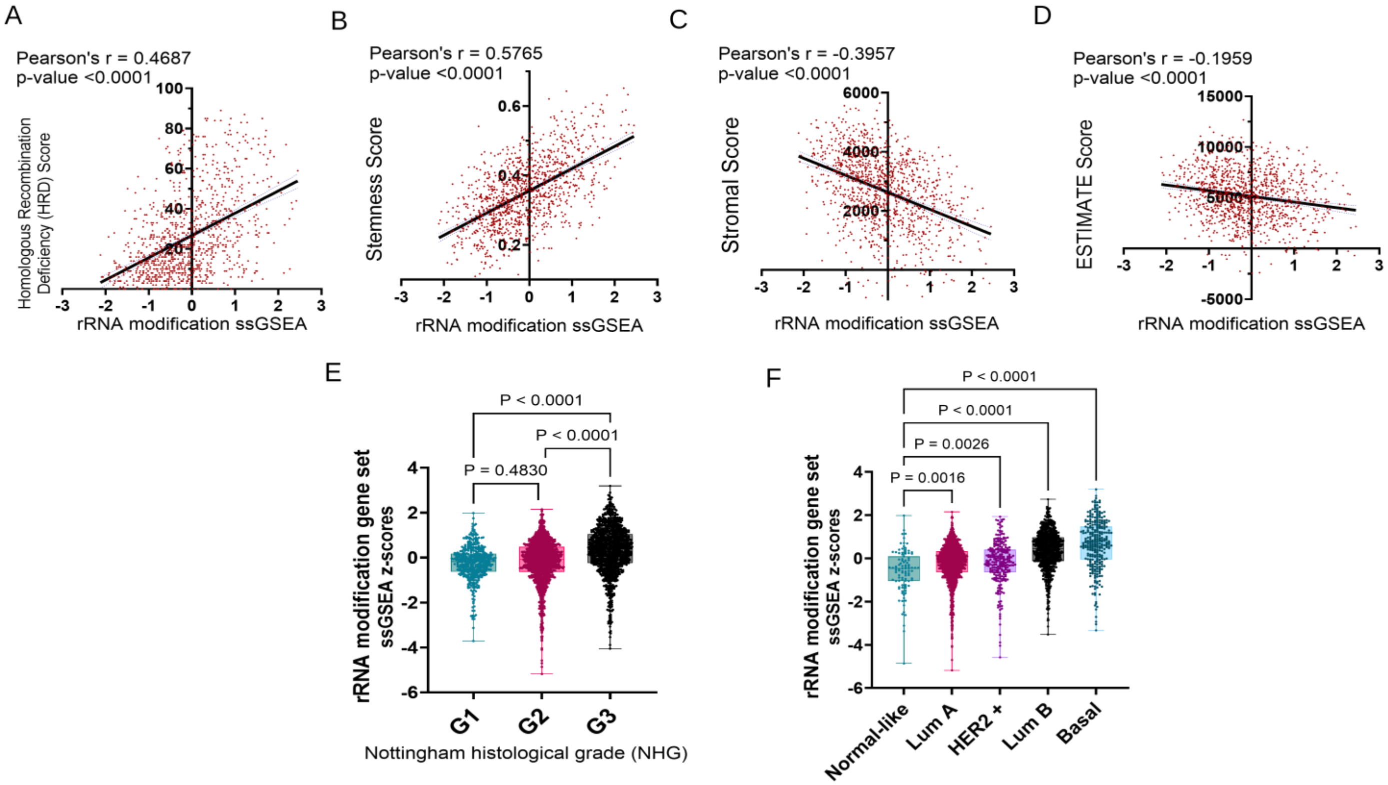

Supp Figure 2. Elevated RRMPs Signature is Linked to Genomic Instability and Tumor Aggressiveness in Breast Cancer

(A) Scatter plot showing a positive correlation between RRMPs signature enrichment (ssGSEA scores) and Homologous Recombination Deficiency (HRD) scores in TCGA breast cancer samples (Pearson's r = 0.4687, p < 0.0001), indicating a link between elevated RRMPs activity and genomic instability.

(B) Scatter plot demonstrating a positive correlation between RRMPs signature enrichment and tumor Stemness Scores (Pearson's r = 0.5765, p < 0.0001), suggesting higher RRMPs activity is associated with increased tumor stemness.

(C) Scatter plot revealing a negative correlation between RRMPs signature enrichment and Stromal Scores (Pearson's r = -0.3957, p < 0.0001), indicating that tumors with elevated RRMPs activity have reduced stromal content.

(D) Scatter plot showing a weak negative correlation between RRMPs signature enrichment and ESTIMATE Scores (Pearson’s $r = -0.1959$, $p < 0.0001$), suggesting that higher RRMPs activity is modestly associated with decreased immune and stromal components in the tumor microenvironment.
(E) Boxplot comparing RRMPs signature enrichment across Nottingham Histological Grades (NHG) using GSE202203 data. RRMPs enrichment significantly increases from G1 to G3, with G3 tumors showing the highest levels, indicating an association between elevated RRMPs activity and tumor aggressiveness.
(F) Boxplot illustrating RRMPs signature enrichment across PAM50 breast cancer subtypes using GSE202203 data. The basal subtype exhibits the highest RRMPs enrichment, underscoring the link between elevated RRMPs activity and aggressive breast cancer phenotypes.

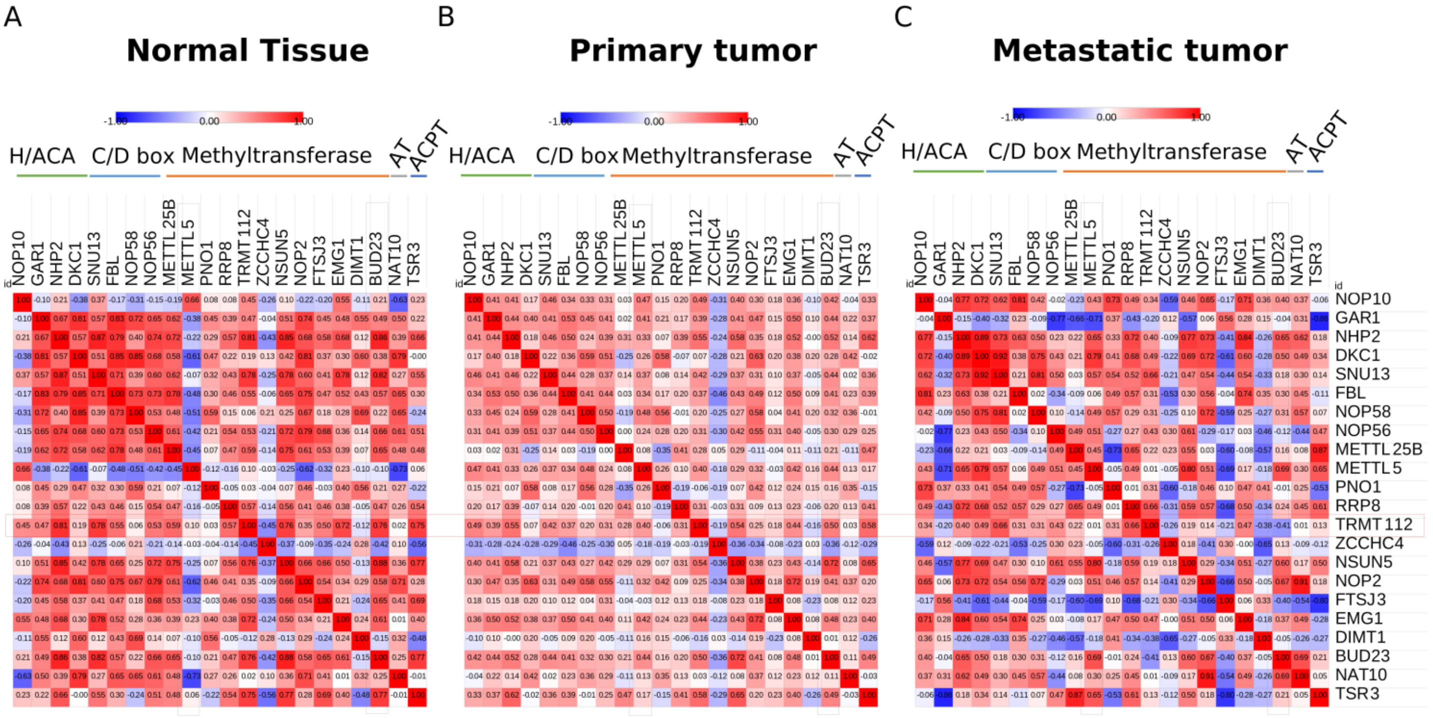

Suppl Figure 3. Distinct Patterns of RRMPs Expression Across Normal, Tumor, and Metastatic Tissues

(A) RRMPs Expression in Normal Tissues: The similarity matrix reveals a highly uniform expression pattern of RRMPs, characterized by strong positive correlations (red) across most proteins. This indicates a well-regulated RRMPs network under normal physiological conditions, ensuring the stability of ribosomal RNA modification processes.

(B) RRMPs Expression in Primary Tumors: The correlation matrix shows increased variability in RRMPs expression compared to normal tissues, reflecting dysregulation in rRNA modification processes associated with tumorigenesis. While some correlations remain intact, others weaken. Notably, METTL5, BUD23, and TRMT112 maintain positive correlations in tumors, whereas METTL25B and TSR3 exhibit reduced

connectivity with other RRMPs, suggesting altered regulatory mechanisms within the tumor microenvironment.

(C) RRMPs Expression in Metastatic Tissues: The metastatic matrix demonstrates a further decline in correlation strength, with the emergence of both positive and negative correlations. This indicates extensive reprogramming of RRMPs networks, potentially enhancing cellular plasticity and adaptability in metastatic progression. The loss of uniformity and the presence of negative correlations suggest significant alterations in the rRNA modification machinery, which may contribute to the survival and spread of metastatic cells in diverse microenvironments.

Similarity matrices were generated using Morpheus (https://software.broadinstitute.org/morpheus), applying Pearson correlation coefficients to assess RRMPs expression relationships across normal, tumor, and metastatic tissues.

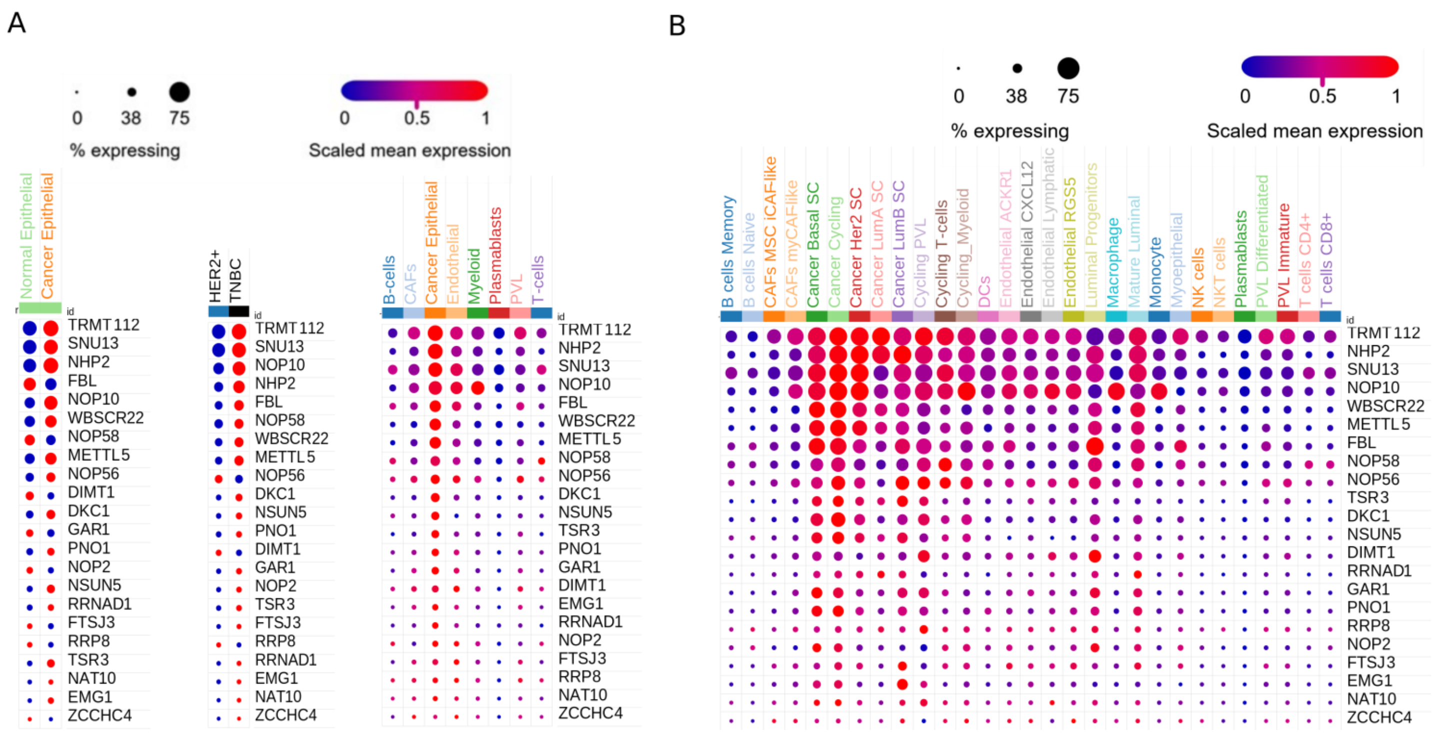

Supp Figure 4. Single-Cell RNA Sequencing Analysis of rRNA Modification-Associated Genes in Breast Cancer.

(A-B) Dot plot illustrating the scaled mean expression and percentage of cells expressing rRNA modification-related genes across diverse cell populations in human breast cancer samples, based on single-cell RNA sequencing (scRNA-seq) data from GSE176078. Data were processed and visualized using the Single Cell Portal (Broad Institute). (A) Expression profiles across normal and malignant epithelial cells, as well as HER2+ and TNBC tumor cells, stromal, and immune compartments. Key ribosome biogenesis regulators, including TRMT112, SNU13, and NOP10, exhibit distinct expression patterns, suggesting differential engagement of translational machinery in specific breast cancer subtypes. (B) Expression and prevalence of selected genes across detailed immune subpopulations, including B cells, T cells, macrophages, and stromal cells, are shown. The color intensity represents the scaled mean expression

(red = high, blue = low), while dot size indicates the percentage of cells expressing each gene. These data reveal context-specific expression of ribosome biogenesis factors, underscoring their potential role in tumor-immune interactions and microenvironmental heterogeneity in breast cancer.

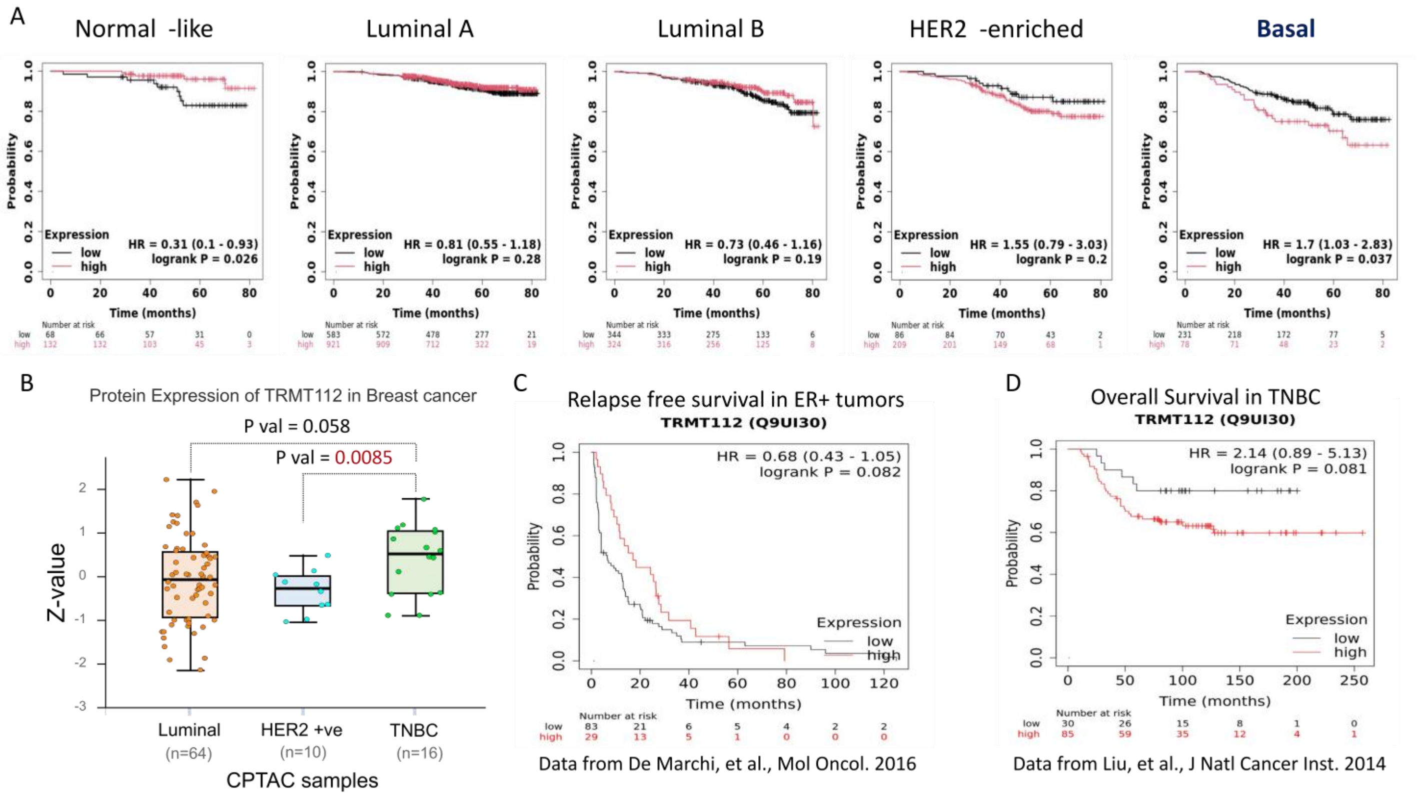

Supp Figure 5. High Levels of TRMT112 are Associated with Poor Prognosis in TNBC

(A) Kaplan-Meier curves illustrate overall survival across breast cancer subtypes based on TRMT112 expression using KMplot data. Elevated TRMT112 is significantly associated with poorer survival in the Basal subtype (HR = 1.7, 95% CI: 1.03–2.83, p = 0.037), while no significant differences are observed in Luminal A, Luminal B, or HER2-enriched subtypes, indicating a subtype-specific prognostic impact.

(B) Boxplot comparing TRMT112 protein levels across Luminal, HER2-positive, and Triple-Negative Breast Cancer (TNBC) subtypes using CPTAC data via the UALCAN platform. TRMT112 expression is significantly higher in TNBC compared to HER2-positive (p = 0.0085) and marginally higher than in Luminal samples (p = 0.058), suggesting its potential as a therapeutic target in TNBC.

(C) Kaplan-Meier plot shows relapse-free survival in estrogen receptor-positive (ER+) breast cancer patients. Although not statistically significant (log-rank $p = 0.082$), the data suggests that higher TRMT112 protein level (red curve) may be associated with a trend towards improved relapse-free survival, with a hazard ratio (HR) of 0.68 (95% CI: 0.43 to 1.05).

(D) Kaplan-Meier plot of overall survival in TNBC patients reveals a contrasting trend, where elevated TRMT112 protein levels (red) are associated with poorer overall survival (HR = 2.14, 95% CI: 0.89–5.13, $p = 0.081$), highlighting TRMT112's context-dependent role in breast cancer progression.

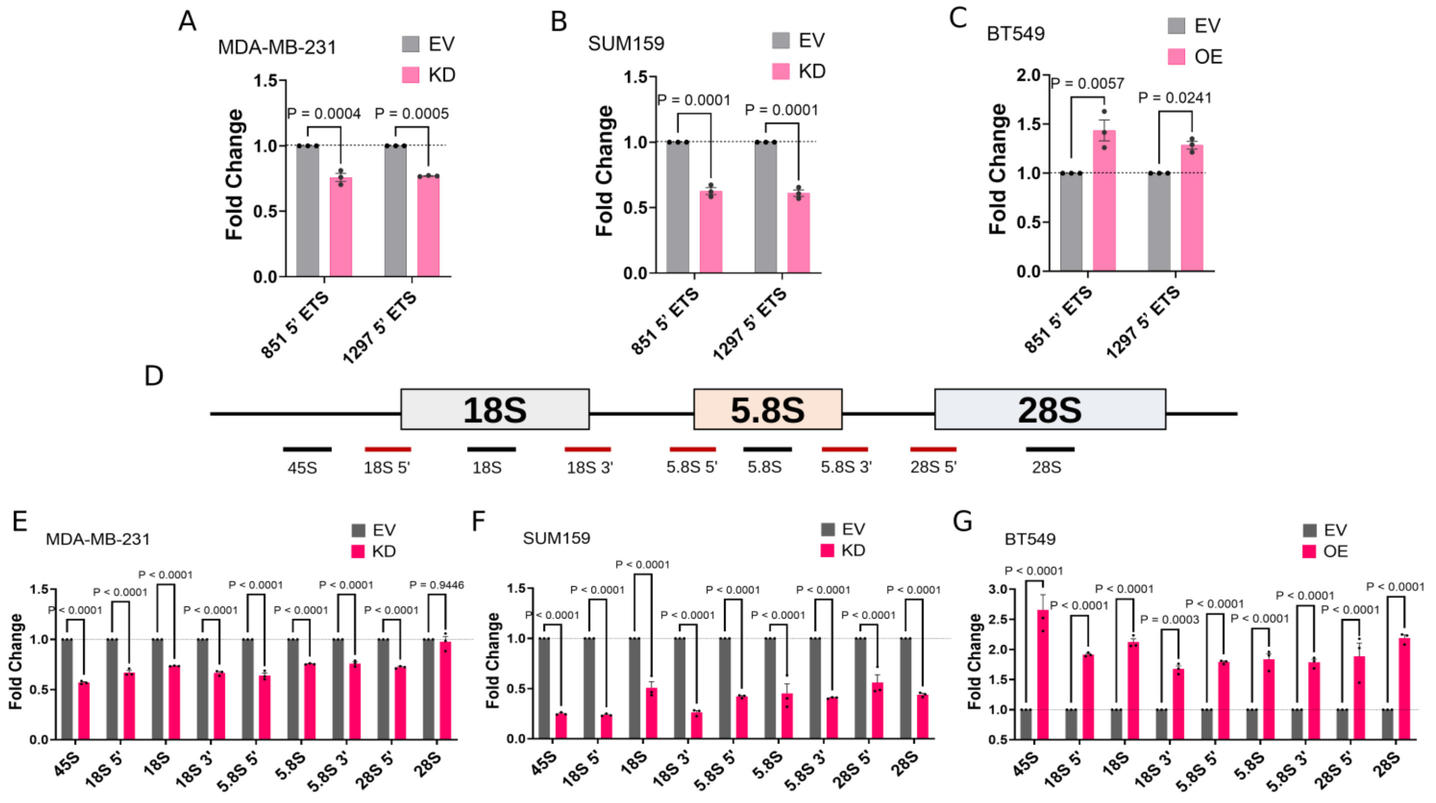

Supp Figure 6. TRMT112 modulates Pol I activity and rRNA processing in breast cancer cells

(A–C) Quantification of RNA Polymerase I (Pol I) activity in TRMT112-modulated breast cancer cells. MDA-MB-231 (A) and SUM159 (B) cells with TRMT112 knockdown (KD) display significantly reduced transcription of 5′ external transcribed spacer (ETS) regions (851 and 1297), compared to empty vector (EV) controls. (C) BT549 cells with TRMT112 overexpression (OE) exhibit increased 5′ ETS transcript levels relative to EV controls. Pre-rRNA levels were normalized to β-actin, and fold-change was calculated relative to empty vector (EV) controls; p-values determined by unpaired two-tailed Student’s t-test.

(D) Schematic representation of human rDNA operon organization, indicating primer sets used to assess rRNA processing intermediates at different positions across the 45S precursor transcript (adapted from Kwon et al., Science 2014).

(E–G) Analysis of rRNA processing intermediates in TRMT112-modulated cells. MDA-MB-231 (E) and SUM159 (F) TRMT112 KD cells show widespread decreases in precursor and intermediate transcripts (45S, 18S 5′, 18S, 18S 3′, 5.8S 5′, 5.8S, 5.8S 3′, 28S 5′), relative to EV controls. (G) Conversely, BT549 TRMT112 OE cells display significantly elevated rRNA processing intermediates compared to EV controls. rRNA intermediates levels were normalized to β-actin, and fold-change was calculated relative to empty vector (EV) controls; statistical significance was determined by unpaired two-tailed Student's t-test.

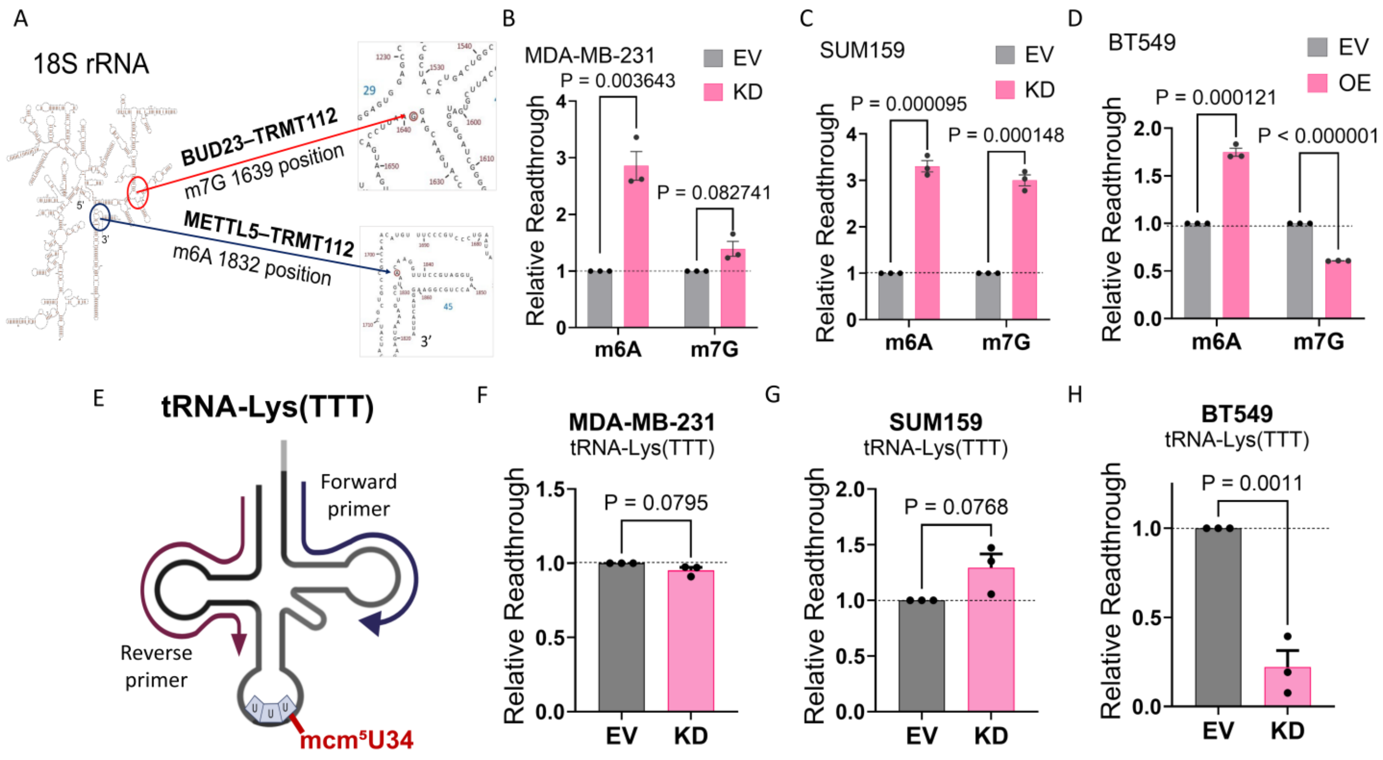

Supp Figure 7. TRMT112 regulates 18S rRNA methylation but has minimal impact on tRNA modification

(A) Schematic representation of TRMT112-associated methylation sites in the 18S rRNA. TRMT112 interacts with BUD23 to catalyze m7G modification at position 1639 and with METTL5 to mediate m6A modification at position 1832.

(B–D) Relative readthrough analysis at 18S rRNA methylation sites in TRMT112-modulated breast cancer cells. TRMT112 knockdown (KD) in MDA-MB-231 (B) and SUM159 (C) cells results in increased readthrough at m6A and m7G sites, indicating reduced modification. In contrast, TRMT112 overexpression (OE) in BT549 cells (D) decreases readthrough at m7G, consistent with enhanced modification. Values >1 indicate weaker pausing and hypomodification, whereas values <1 indicate stronger pausing and higher modification levels. rRNA readthrough values were normalized to β-

actin, and fold-change was calculated relative to EV controls. Statistical significance was determined by unpaired two-tailed Student's t-test.

(E) Schematic of the tRNA-Lys(TTT) secondary structure showing the mcm^5U34 modification site and primer locations used for readthrough analysis.

(F–H) Relative readthrough at the mcm^5U34 site of tRNA-Lys(TTT). TRMT112 knockdown in MDA-MB-231 (F) and SUM159 (G) cells does not significantly affect readthrough. TRMT112 OE in BT549 cells (H) reduces readthrough, consistent with increased modification; however, these effects were not consistently significant across cell lines, suggesting that TRMT112 primarily impacts rRNA methylation rather than tRNA modification. tRNA readthrough values were normalized to U6 snRNA and fold-change calculated relative to EV controls. Statistical significance was determined by unpaired two-tailed Student's t-test.

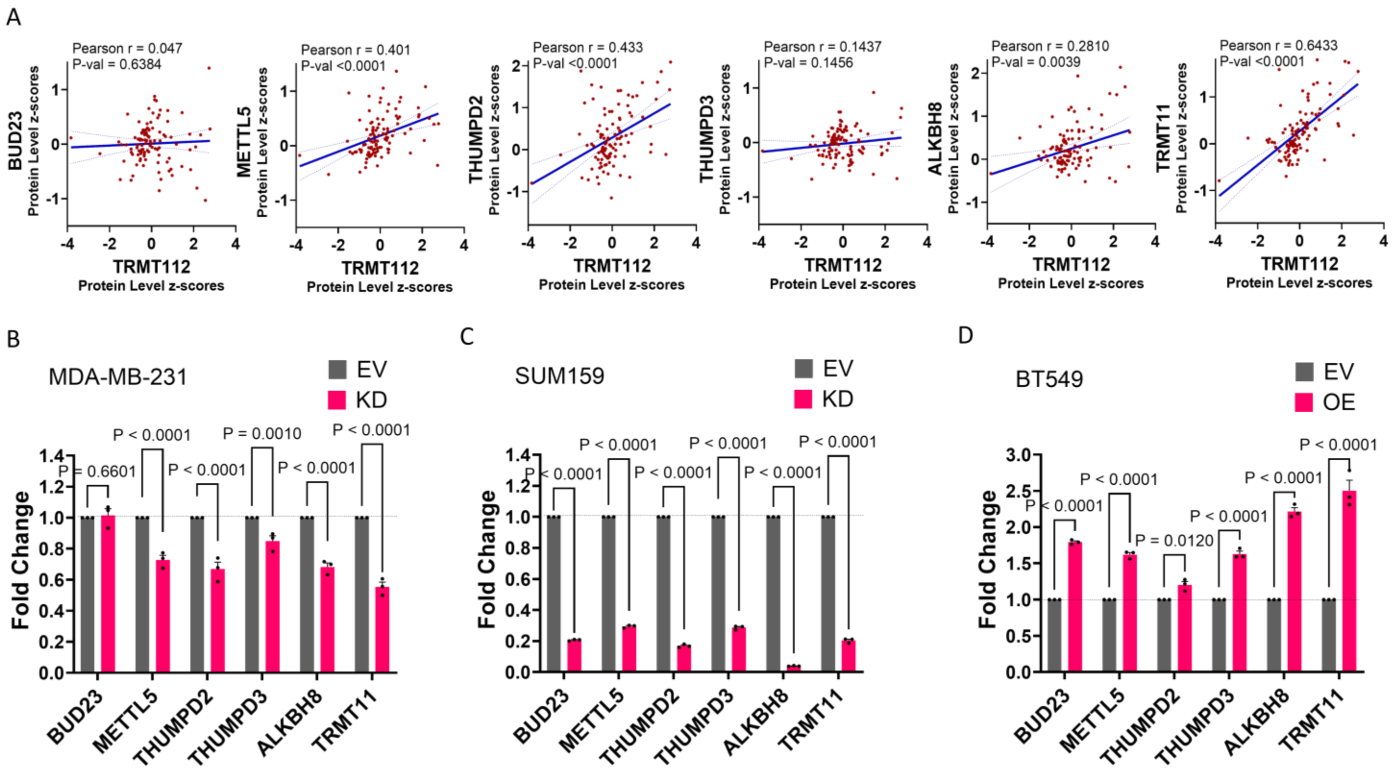

Supp Figure 8. TRMT112 modulation alters the expression of its interacting partners and correlates with their protein levels in breast cancer

(A) Correlation analysis between TRMT112 protein levels and its cofactors in the TCGA-BRCA CPTAC dataset (n= 104 samples). Protein z-scores for BUD23, METTL5, THUMPD2, THUMPD3, TRMT11, and ALKBH8 were compared against TRMT112 protein levels. Pearson correlation coefficients and corresponding p-values are shown. Significant positive correlations were observed for METTL5, THUMPD2, TRMT11, and ALKBH8, whereas correlations with BUD23 and THUMPD3 were not significant.

(B–D) qPCR analysis of TRMT112 cofactors and interacting partners (BUD23, METTL5, THUMPD2, THUMPD3, ALKBH8, and TRMT11) in TRMT112-modulated breast cancer cells. Knockdown (KD) of TRMT112 in MDA-MB-231 (A) and SUM159 (B) cells significantly reduces transcript levels of several cofactors, while overexpression (OE) of

TRMT112 in BT549 cells (C) enhances their expression. Data are presented as fold-change relative to empty vector (EV) controls, with β-actin used as the housekeeping/internal control. P-values were determined by unpaired two-tailed Student’s t-test.

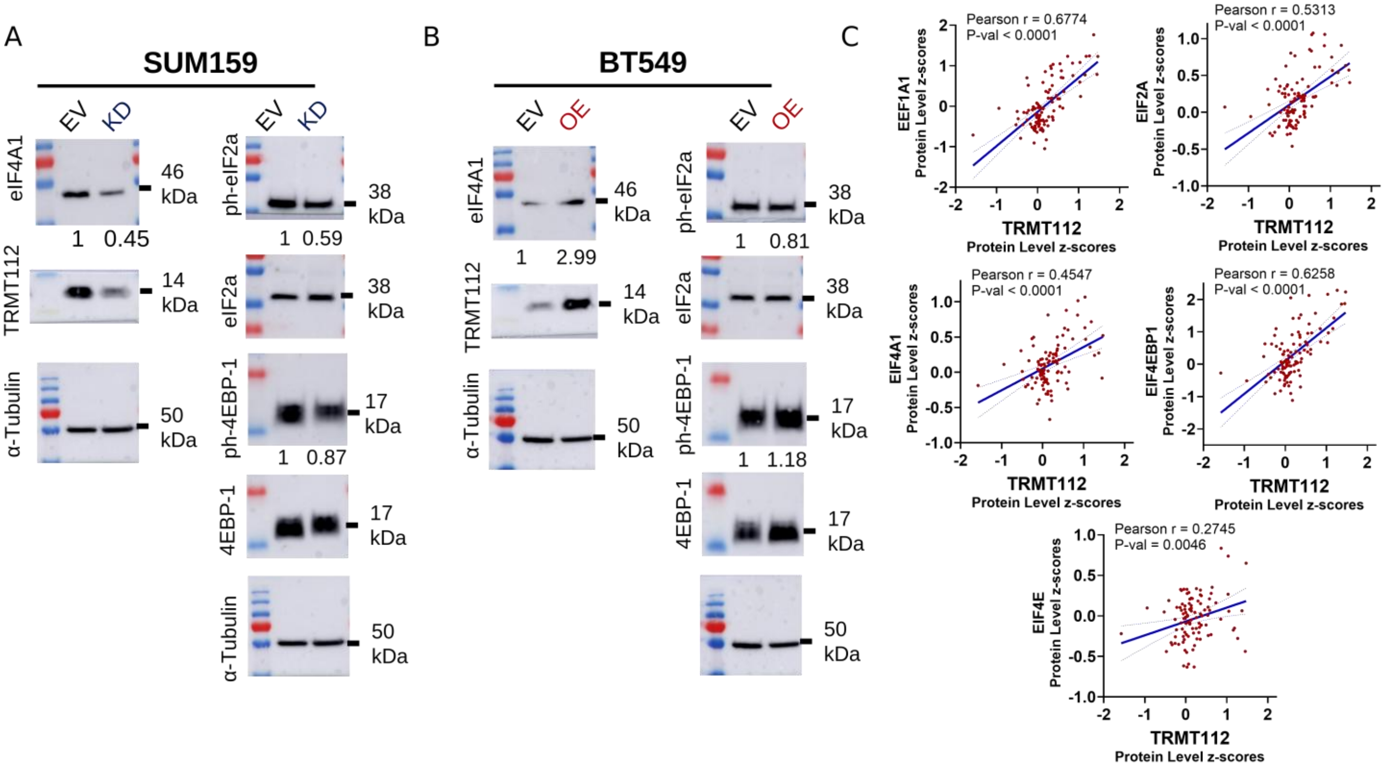

Supp Figure 9. TRMT112 modulates translation factors abundance and phosphorylation in breast cancer cells

(A–B) Immunoblot analysis of key translation factors in TRMT112-modulated breast cancer cells. TRMT112 knockdown (KD) in SUM159 cells (A) reduces levels of eIF4A1 and phosphorylated eIF2α (ph-eIF2α), while TRMT112 overexpression (OE) in BT549 cells (B) increases eIF4A1 abundance. Phosphorylation of eIF2α and 4EBP-1 was quantified relative to their corresponding total protein levels. α-Tubulin served as the loading control. Densitometric quantification was normalized to α-tubulin for total protein abundance and expressed relative to untreated empty vector (EV) controls (set to 1). All blots were generated from a single set of cell lysates, which was probed for the factors shown.

(C) Correlation analysis between TRMT112 protein levels and several translation factors in the TCGA-BRCA CPTAC dataset (n= 104 samples). Significant positive correlations

were observed between TRMT112 and eEF1A1, eIF4A1, eIF2A, 4E-BP1 and eIF4E protein levels, with Pearson correlation coefficients and p-values indicated for each.

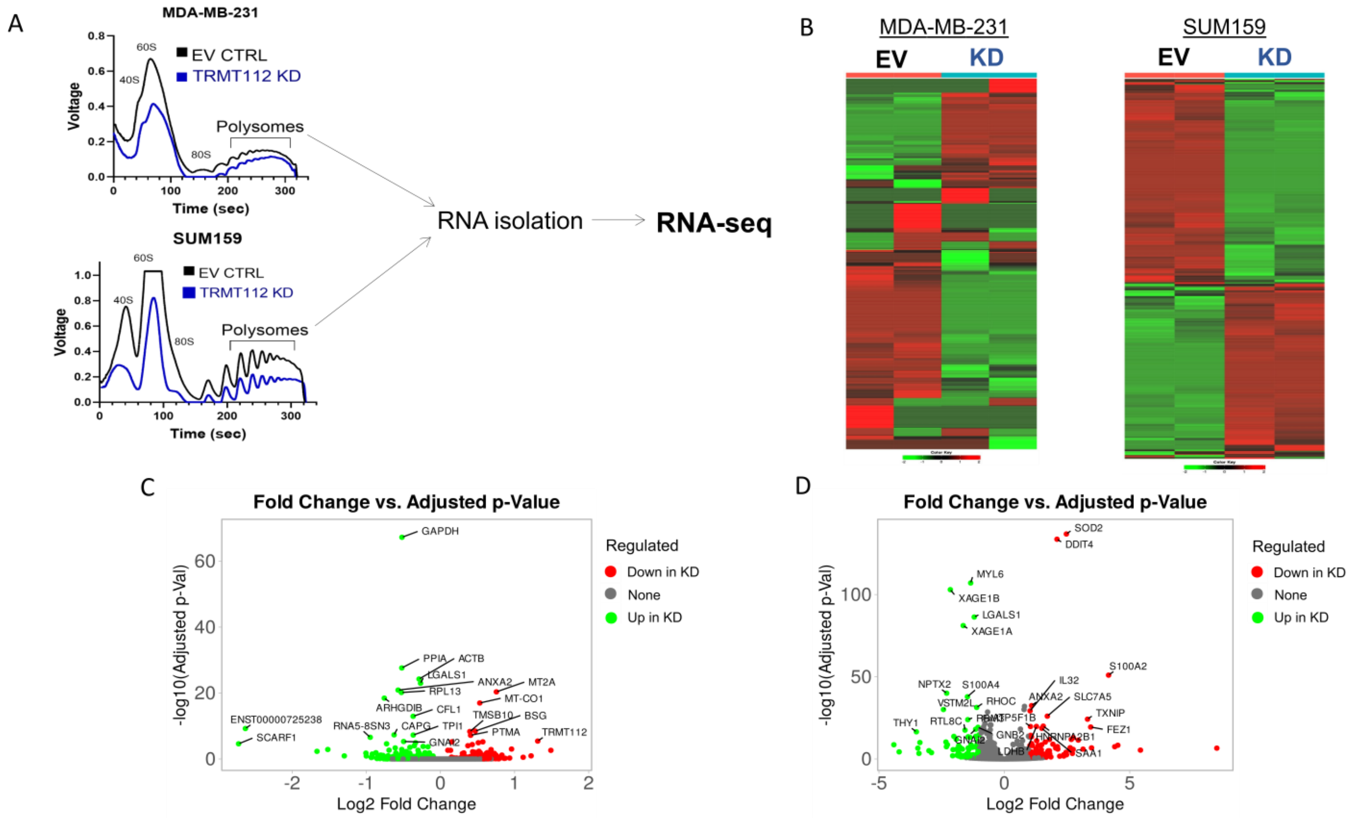

Supp Figure 10. TRMT112 Knockdown Reduces Polysome Formation and Alters the Actively Translated mRNA Pool in TNBC Cells

(A) Polysome-profiling RNA-seq workflow. Sucrose-gradient absorbance profiles from MDA-MB-231 and SUM159 cells expressing either empty vector (EV, black) or TRMT112 shRNA (KD, blue). TRMT112 depletion reduces 80S and polysome peaks, indicating lower global ribosome loading. Pooled polysome fractions from the same gradients were subjected to RNA-seq to define TRMT112-dependent changes in the actively translated transcriptome.

(B) Hierarchical clustering heatmaps displaying transcriptomic changes in polysome-associated mRNAs from MDA-MB-231 (left) and SUM159 (right) cells after TRMT112 knockdown. Upregulated transcripts are shown in red, while downregulated transcripts are in green, highlighting widespread translational reprogramming.

(C) A volcano Plot displays changes in the actively translated transcriptome of MDA-MB-231 cells after TRMT112 KD. Each point represents a transcript, plotted by its $\log_2$ fold change in polysome association (KD versus EV) on the x-axis and the $-\log_{10}$ adjusted P value on the y-axis. Transcripts whose association with polysomes is significantly reduced in TRMT112 KD cells are colored red, whereas those that exhibit greater polysome enrichment, and hence higher translation, are colored green; transcripts that are not differentially enriched in polysomes are grey.

(D) A volcano plot illustrates changes in the actively translated transcriptome upon TRMT112 knockdown in SUM159 cells. Transcripts with reduced polysome association in TRMT112 KD cells are red, those with increased association are green, and unchanged transcripts are grey.

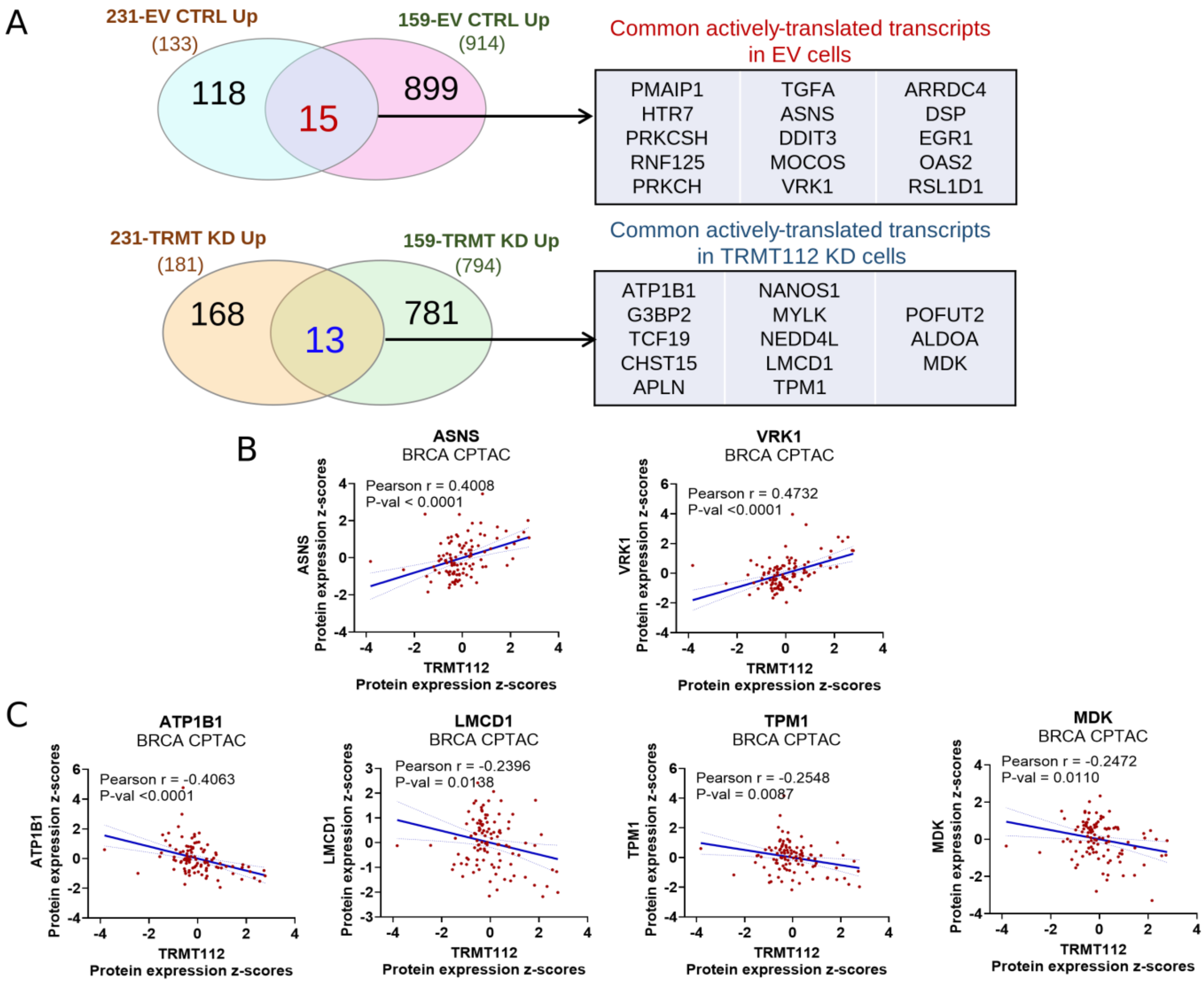

Supp Figure 11. TRMT112 knockdown reprograms the actively translated transcriptome to suppresses metastatic and invasive signatures in TNBC cells

(A) Venn diagrams illustrate differentially translated genes unique to each condition and shared between the two cell lines. TRMT112 KD cells exhibit a distinct translational landscape compared to EV cells.

(B) Scatter plots showing a significant positive correlation between TRMT112 expression and the protein levels of ASNS and VRK1 in BRCA CPTAC datasets, indicating that these genes are preferentially translated in EV cells. These genes are

linked to cell survival, proliferation, and stress response, suggesting that TRMT112 supports a pro-tumorigenic translation program.

(C) Scatter plots demonstrating a significant inverse correlation between TRMT112 expression and ATP1B1, LMCD1, TPM1, and MDK protein levels in BRCA CPTAC datasets, suggesting that these genes are actively translated in TRMT112 KD cells. Many of these genes are associated with differentiation, adhesion, and cytoskeletal remodeling, consistent with the reduced metastatic and invasive phenotype observed in TRMT112 KD cells.

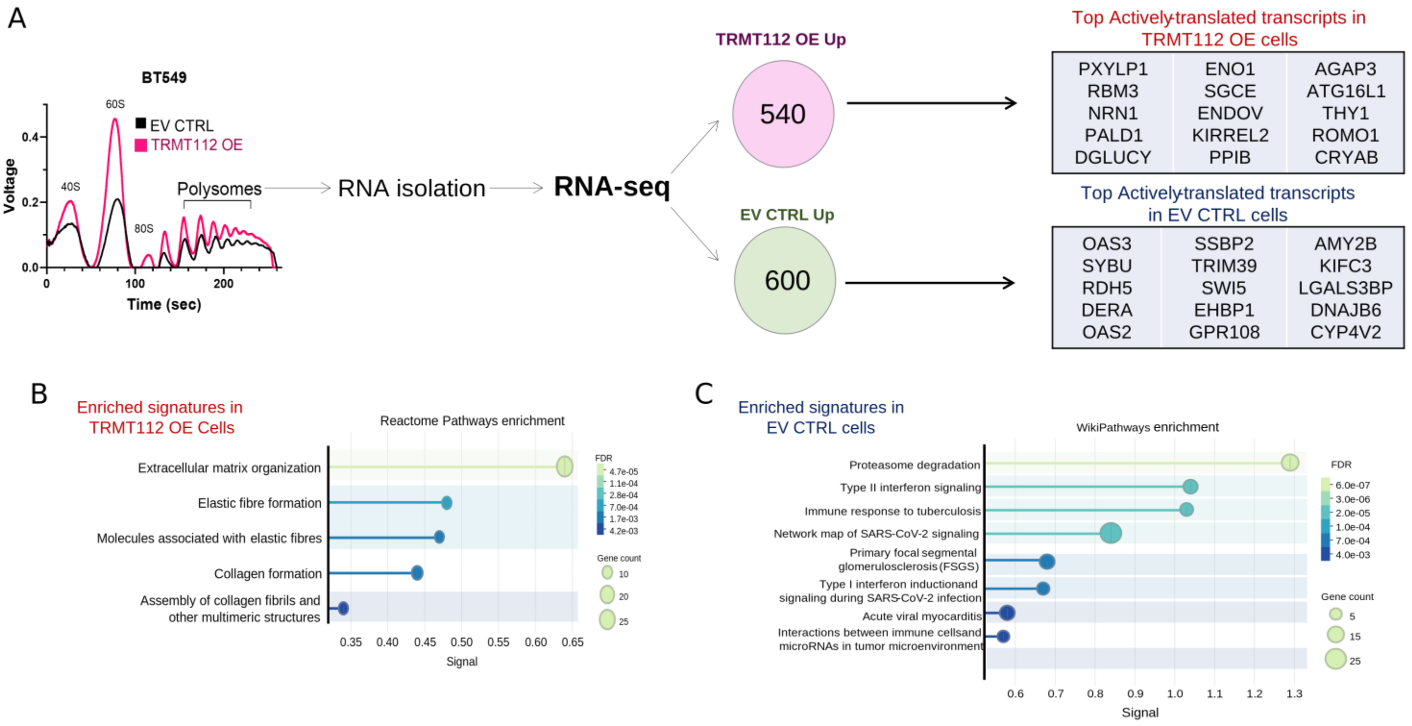

Supp Figure 12. TRMT112 overexpression reprograms the actively translated transcriptome to enhance metastatic potential and immune evasion in TNBC cells.

(A) Polysome profiling was performed on EV and TRMT112 OE BT549 cells to isolate actively translated mRNAs, followed by RNA sequencing (RNA-seq). Venn diagrams illustrate the distinct sets of actively translated genes in each condition.

(B) STRING-db analysis showing that TRMT112 overexpression enhances translation of genes involved in stromal organization, and collagen formation, processes linked to increased matrix stiffening and metastatic potential.

(C) STRING-db analysis revealing that EV cells are enriched in immune-related pathways, interferon signaling, immune response, and cytokine signaling, indicative of an anti-tumor immune response. These findings suggest that TRMT112 overexpression

promotes a pro-metastatic translational program while inhibiting immune-related pathways.

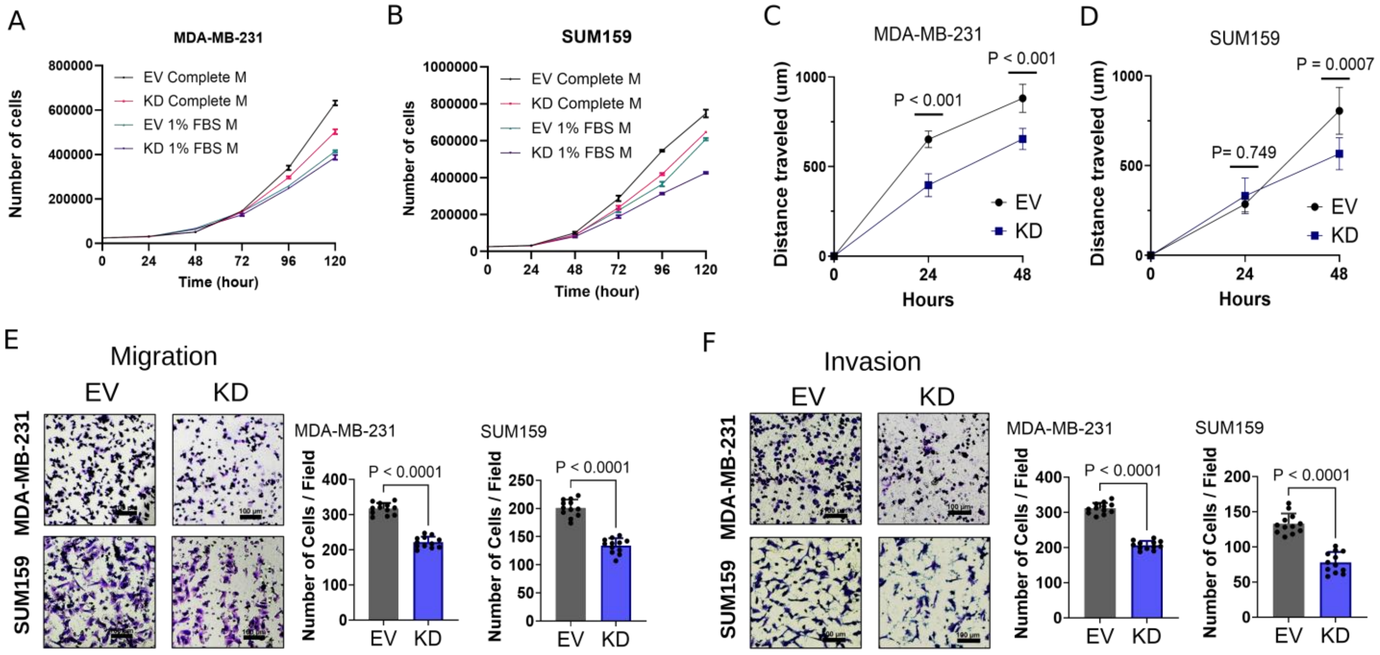

Supp Figure 13. TRMT112 knockdown impairs proliferation, migration, and invasion in TNBC cells

(A-B) Cell growth curves for MDA-MB-231 and SUM159 cells over 5 days, comparing TRMT112 knockdown (KD) and empty vector (EV) cells across both complete media and 1% FBS media conditions, demonstrating minimal differences in proliferation.

(C-D) Wound healing assays reveal that TRMT112 knockdown significantly reduces cell migration (distance traveled) in MDA-MB-231 cells (C) at both 24 and 48 hours ($p < 0.001$), while SUM159 cells (D) show a significant decrease only at 48 hours ($p = 0.0007$), indicating TRMT112's role in promoting cellular motility.

(E) Transwell migration assays performed for 4 hours in MDA-MB-231 and SUM159 cells. TRMT112 KD significantly decreases the number of migrating cells compared to EV controls. Representative images (left) and quantification (right) are shown. p-values were determined by unpaired two-tailed Student's t-test. Scale bars = 100 μm.

(F) Matrigel invasion assays performed for 12 hours in MDA-MB-231 and SUM159 cells. TRMT112 KD significantly reduces invasive capacity relative to EV controls. Representative images (left) and quantification (right) are shown. p-values were determined by unpaired two-tailed Student’s t-test. Scale bars = 100 μm.

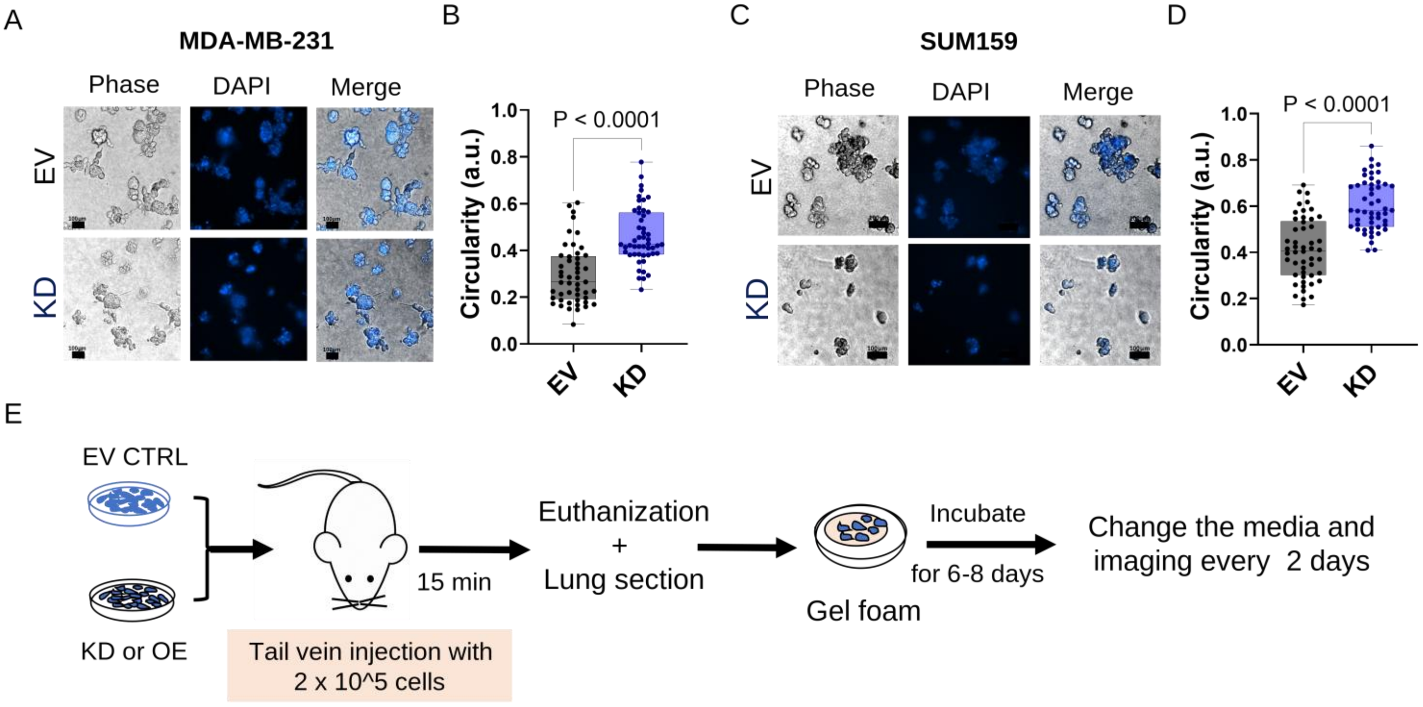

Supp Figure 14. TRMT112 Knockdown Impairs Invasiveness in TNBC cells

(A-D) 3D culture of TRMT112 KD in MDA-MB-231 (A) and SUM159 (C) cells display morphological differences compared to controls. The KD cells have less spikes and show increased circularity as shown in (B) and (D), respectively, suggesting a decrease in invasive potential. This panel supports the notion that TRMT112 contributes to the aggressive and invasive characteristics of breast cancer cells. Scale bars = 100 μm. (E) A schematic illustrates the procedure for assessing the influence of TRMT112 on breast cancer metastasis. Cells either overexpressing TRMT112 or with TRMT112 knocked down, alongside empty vector controls, were injected into mice. Following a circulation period, the mice were euthanized, and lung tissues were harvested and cultured on gel foam for imaging every two days over 4-7 days to observe metastatic colonization.

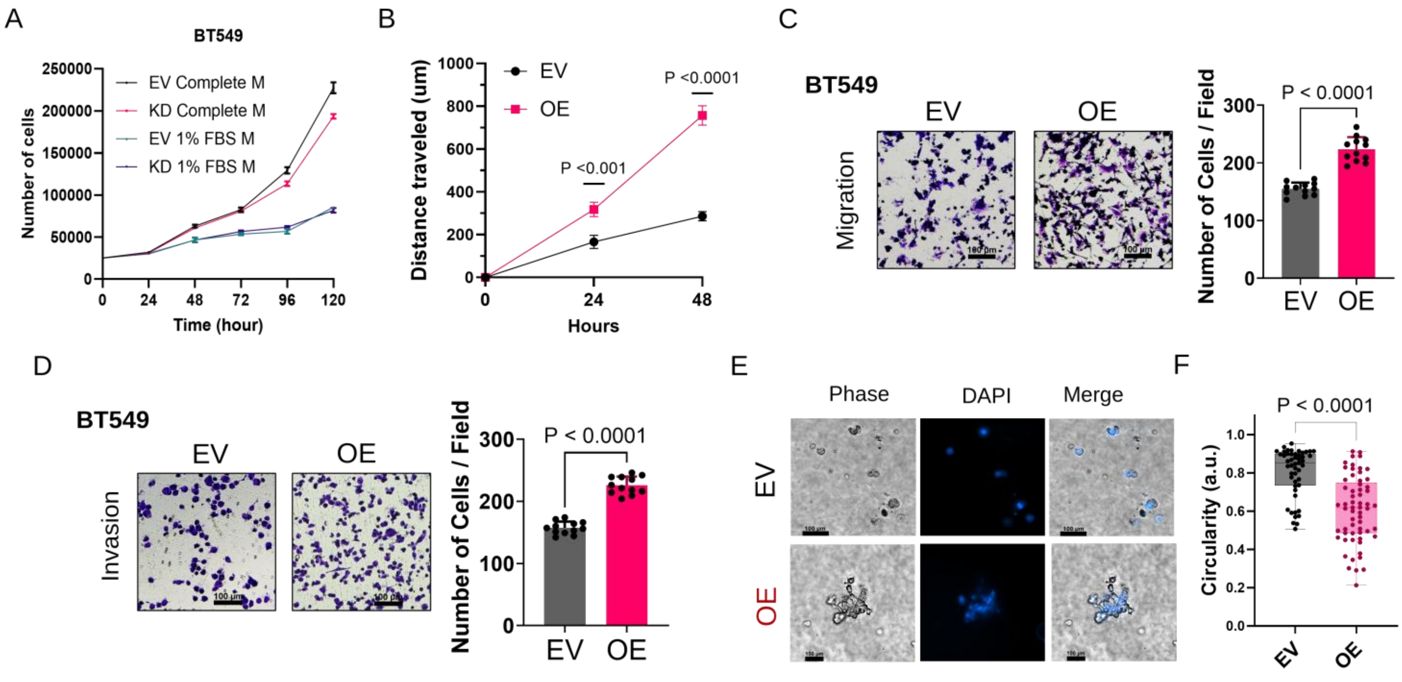

Supp Figure 15. TRMT112 overexpression enhances proliferation, migration, invasion, and 3D growth in TNBC Cells

(A) Cell growth curve in BT549 cells with TRMT112 overexpression (OE) versus EV cells, assessed in both complete medium and low serum (1% FBS) conditions.

(B) A wound healing assay shows that TRMT112 overexpression in BT549 cells significantly enhances migration, with greater distances traveled at 24 and 48 hours compared to controls ($p < 0.001$ and $p < 0.0001$, respectively).

(C) Transwell migration assays performed over 4 hours. TRMT112 OE cells exhibit a significantly higher number of migrating cells compared to EV controls. Representative images (left) and quantification (right) are shown. p-values were determined by unpaired two-tailed Student's t-test. Scale bars = 100 µm.

(D) Matrigel invasion assays performed over 12 hours. TRMT112 OE significantly enhances invasive capacity relative to EV controls. Representative images (left) and

quantification (right) are shown. p-values were determined by unpaired two-tailed Student’s t-test. Scale bars = 100 μm.

(E–F) 3D culture assays showing altered morphology of TRMT112 OE spheroids compared to EV controls. Representative images of phase-contrast and DAPI staining (E) demonstrate increased cellular clustering in OE spheroids. Quantification of spheroid circularity (F) indicates that TRMT112 OE significantly decreases circularity, consistent with a more invasive 3D growth phenotype. p-values were determined by unpaired two-tailed Student’s t-test. Scale bars = 100 μm.

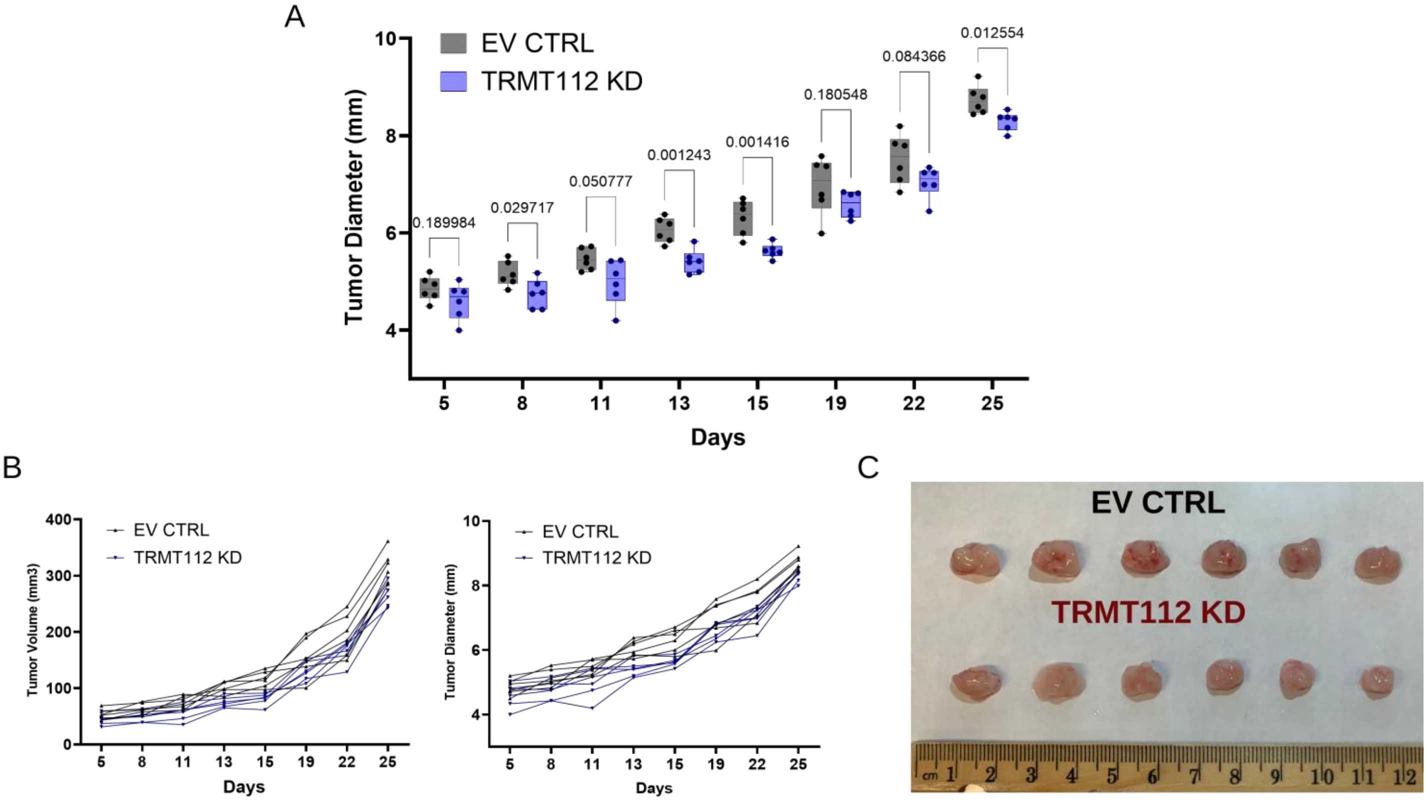

Supp Figure 16. TRMT112 knockdown suppresses primary tumor growth and metastatic progression in an orthotopic breast cancer model.

(A) Tumor diameter measurements at the indicated time points. Data represent mean ± SD of measurements from 6 mice per group. Statistical significance was determined using a two-tailed Student's t-test; p-values are indicated for each time point.

(B) Growth curves of tumor volume (left) and tumor diameter (right) in EV CTRL and TRMT112 KD groups over time. Individual mouse data are plotted, showing slower tumor growth in the TRMT112 KD group compared to the EV CTRL group.

(C) Representative images of primary tumors excised from NSG mice orthotopically injected with either EV or TRMT112 KD cells.
