# Supplementary material for: TRMT112 drives a tumor growth and metastasis-promoting program in triple-negative breast cancer
Source: Cell Death Differ. 2026 Jan 8;33(6):1192–202. doi: 10.1038/s41418-025-01643-z (PMC13246786; doi:10.1038/s41418-025-01643-z)
Supplement: Supplementary file 3 — Related Manuscript File [file 41418_2025_1643_MOESM3_ESM.pdf]

Key Resources Table

Antibodies

REAGENT or RESOURCE	SOURCE	IDENTIFIER
Anti-Puromycin	Sigma-Aldrich	Cat# MABE343
Anti-α-tubulin	Cell Signaling Technologies	Cat# 3873S
anti-β-actin	Sigma-Aldrich	Cat# A3854
Anti-TRMT112 antibody	Sigma-Aldrich	Cat# HPA040006
Anti-rabbit HRP-conjugated secondary antibody	GE Healthcare	Cat# NA934
Anti-mouse HRP-conjugated secondary antibody	GE Healthcare	Cat# NA931
Anti-eIF4A1 antibody	Cell Signaling Technologies	Cat# 2490
Anti-ph-eIF2α antibody	Cell Signaling Technologies	Cat# 3398
Anti-4EBP-1 antibody	Cell Signaling Technologies	Cat# 9644
Anti-ph-4EBP-1 antibody	Cell Signaling Technologies	Cat# 9451
Anti-eIF2a antibody	Cell Signaling Technologies	Cat#2103

Cell Lines

CELL LINE	SOURCE	IDENTIFIER
MDA-MB-231	ATCC	Cat# HTB-26
SUM159	Asterand Bioscience	NA
BT-549	ATCC	Cat# HTB-122
MCF10A	ATCC	Cat# CRL-10317
SKBr3	ATCC	Cat# HTB-30
SUM149	Asterand Bioscience	NA
HMEC	Lonza	Cat# CC-2551

HMLE	ATCC	Cat# CRL-3063
BT-474	ATCC	Cat# HTB-20
SUM1315	Asterand Bioscience	NA
ZR-75-30	ATCC	Cat# CRL-1504
MCF-7	ATCC	Cat# HTB-22
MCF7-5624	ATCC	NA

Cell Culture Reagents

REAGENT	SOURCE	IDENTIFIER
RPMI 1640 Media	Thermo Fisher Scientific	Cat# 22400105
DMEM/F-12 Media	Thermo Fisher Scientific	Cat# 11330032
Fetal Bovine Serum (FBS)	Thermo Fisher Scientific	Cat# A5256701
Dulbecco's Phosphate-Buffered Saline (DPBS)	Thermo Fisher Scientific	Cat# 14190144
Puromycin	Sigma-Aldrich	Cat# P8833
G418 Sulfate	Thermo Fisher Scientific	Cat# 10131027
Lipofectamine™ 2000 Transfection Reagent	Thermo Fisher Scientific	Cat# 11668027
Cycloheximide	Sigma-Aldrich	Cat# 239764-1GM
Halt™ Protease Inhibitor Cocktail	Thermo Fisher Scientific	Cat# 78442

Gene Manipulation Reagents

REAGENT	SOURCE	IDENTIFIER
GIPZ TRMT112 Lentiviral shRNA	Horizon Discovery	Cat# RHS4531-EG51504
GIPZ Non-silencing Lentiviral shRNA	Horizon Discovery	Cat# RHS4346
TRMT112 ORF Expression Clone	GeneCopoeia	Cat# EX-J0039-M02-B
pReceiver-M02 Empty Vector	GeneCopoeia	Cat# EX-NEG-M02-B

3D Culture Reagents

REAGENT	SOURCE	IDENTIFIER

Cultrex 3D Culture Matrix Reduced Growth Factor	R&D Systems	Cat# 3445-010-01

Imaging Reagents and Equipment

REAGENT / EQUIPMENT	SOURCE	IDENTIFIER
NucBlue™ Live ReadyProbes™ Reagent	Thermo Fisher Scientific	Cat# R37605
Nikon Eclipse Ti-U Microscope	Nikon Instruments Inc	NA
Nikon SMZ800 Stereo Zoom Microscope	Advanced Cell Diagnostics	Cat# 322370
NIS-Elements AR Software	Nikon Instruments Inc	NA

Histological and IHC Staining Reagents

REAGENT	SOURCE	IDENTIFIER
Picrosirius Red Stain Kit	Polysciences	NA

Computational and Bioinformatics Tools

SOFTWARE / TOOL	SOURCE	URL / IDENTIFIER
GraphPad Prism	GraphPad Software	Version 9
Morpheus	Broad Institute	Morpheus
GenePattern	Broad Institute	GenePattern
ImageJ	National Institutes of Health	ImageJ
cBioPortal	Center for Molecular Oncology at MSK	cBioPortal
TCGA Data Portal	National Cancer Institute	TCGA
GEPIA2	NA	GEPIA2
TNMplot Tool	NA	TNMplot

RNA Sequencing and Gene Expression Analysis Tools

TOOL / DATABASE	SOURCE	URL
Gene Expression Omnibus (GEO)	NCBI	GEO
STRING-db	STRING Consortium	STRING-db
DESeq2	Bioconductor	DESeq2
HISAT2	Johns Hopkins University	HISAT2
SAMtools	Broad Institute	SAMtools
Trimmomatic	Usadel Lab	Trimmomatic

Primers and Oligonucleotides

REAGENT or RESOURCE	SOURCE	IDENTIFIER
Human 5’ETS 851-961 Forward primer<br>5'-GAACGGTGGTGTGTCGTT-3'	Peltonen et al. [1]	NA
Human 5’ETS 851-961 Reverse primer<br>5'-GCGTCTCGTCTCGTCTCACT-3'	Peltonen et al. [1]	NA
Human 5’ETS 1297-1483 Forward primer<br>5'-CAGGTGTTTCCTCGTACCG-3'	Peltonen et al. [1]	NA
Human 5’ETS 1297-1483 Reverse primer<br>5'-GCTACCATAACGGAGGCAGA-3'	Peltonen et al. [1]	NA
Human Actin Forward primer<br>5'-CATGTACGTTGCTATCCAGGC-3'	PrimerBank [2]	ID: 4501885a1
Human Actin Reverse primer<br>5'-CTCCTTAATGTCACGCACGAT-3'	PrimerBank [2]	ID: 4501885a1
TRMT112 Forward primer<br>5'-GGTCCGTATCTGCCCTGTG-3'	PrimerBank [2]	ID: 7705476c1
TRMT112 Reverse primer<br>5'-GGATCAGACGCAAGTTATCGG-3'	PrimerBank [2]	ID: 7705476c1
BUD23 Forward primer<br>5'-CCCTGTTACCTGCTGGATATTG-3'	PrimerBank [2]	ID: 356874773c2
BUD23 Reverse primer<br>5'-ATGCAACCATCAAATGTGCCT-3'	PrimerBank [2]	ID: 356874773c2
METTL5 Forward primer<br>5'-AAGGAACTAGAGAGTCGCCTG-3'	PrimerBank [2]	ID: 92859574c1
METTL5 Reverse primer	PrimerBank [2]	ID: 92859574c1

5'-GCGGCCTGGTAGGATACTG-3'		
THUMPD3 Forward primer<br>5'-CCAACTCCTAGATGTGAACCTTC-3'	PrimerBank [2]]	ID: 166197707c1
THUMPD3 Reverse primer<br>5'-AGTGGCTCCAATAGTGACTAGAA-3'	PrimerBank [2]	ID: 166197707c1
THUMPD2 Forward primer<br>5'-CAGCAGAGCTTACATCAAGACA-3'	PrimerBank [2]	ID: 254553430c1
THUMPD2 Reverse primer<br>5'-GTAACTGTGAGTCGCTGACATC-3'	PrimerBank [2]	ID: 254553430c1
ALKBH8 Forward primer<br>5'-ATGGACAGCAACCATCAAAGTAA-3'	PrimerBank [2]	ID: 195927055c1
ALKBH8 Reverse primer<br>5'-GGCTCTGAGTGGCATAGGATAC-3'	PrimerBank [2]	ID: 195927055c1
TRMT11 Forward primer<br>5'-TCCGCCTGCCGGAAATAAAG-3'	PrimerBank [2]	ID: 94420682c1
TRMT11 Reverse primer<br>5'-ACACACTGTCCGTTTCATCAAAT-3'	PrimerBank [2]	ID: 94420682c1
45S- Forward primer<br>5'-GAACGGTGGTGTGTCGTT-3'	Kwon et al. [3]	NA
45S- Reverse primer<br>5'-GCGTCTCGTCTCGTCTCACT-3'	Kwon et al. [3]	NA
18S-5'-junction- Forward primer<br>5'-GCCGCGCTCTACCTTACCTACCT-3'	Kwon et al. [3]	NA
18S-5'-junction- Reverse primer<br>5'-CAGACATGCATGGCTTAATCTTTG-3'	Kwon et al. [3]	NA
18S-3'-junction- Forward primer<br>5'-AGTCGTAACAAGGTTTCCGTAGGT-3'	Kwon et al. [3]	NA
18S-3'-junction- Reverse primer<br>5'-CCTCCGGGCTCCGTTAAT-3'	Kwon et al. [3]	NA
5.8S-5'-junction- Forward primer<br>5'-TACGACTCTTAGCGGTGGATCA-3'	Kwon et al. [3]	NA
5.8S-5'-junction- Reverse primer<br>5'-TCACATTAATTCTCGCAGCTAGCT-3'	Kwon et al. [3]	NA
5.8S-3'-junction- Forward primer<br>5'-GAATTGCAGGACACATTGATCATC-3'	Kwon et al. [3]	NA
5.8S-3'-junction- Reverse primer<br>5'-GGCAAGCGACGCTCAGA-3'	Kwon et al. [3]	NA
28S-5'-junction- Forward primer<br>5'-CCGAGACGCGACCTCAGAT-3'	Kwon et al. [3]	NA
28S-5'-junction- Reverse primer<br>5'-TCCGCTGACTAATATGCTTAAATTCA-3'	Kwon et al. [3]	NA
18S- Forward primer	Kwon et al. [3]	NA

5'-GATGGTAGTCGCCGTGCC-3'		
18S- Reverse primer<br>5'-GCCTGCTGCCTTCCTTGG-3'	Kwon et al. [3]	NA
5.8S- Forward primer<br>5'-ACTCGGCTCGTGCGTC-3'	Kwon et al. [3]	NA
5.8S- Reverse primer<br>5'-GCGACGCTCAGACAGG-3'	Kwon et al. [3]	NA
28S- Forward primer<br>5'-GTGACGCGCATGAATGGA-3'	Kwon et al. [3]	NA
28S- Reverse primer<br>5'-TGTGGTTTCGCTGGATAGTAGGT-3'	Kwon et al. [3]	NA
hs-tRNA-Lys(TTT)- Forward primer<br>5'-TAGCTCAGTCGGTAGAGCAT-3'	This paper	NA
hs-tRNA-Lys(TTT)- Reverse primer<br>5'-TCCAGGGTTCAAGTCCCTGT-3'	This paper	NA
U6 snRNA- Forward primer<br>5’-CTCGCTTCGGCAGCACATA-3'	This paper	NA
U6 snRNA- Reverse primer<br>5’-CGAATTTGCGTGTCATCCT-3'	This paper	NA
18S RNA -1639 m7G- Forward primer<br>5’-GGTAACCCGTTGAACCCCATTCG-3'	This paper	NA
18S RNA -1639 m7G- Reverse primer<br>5’-GCGACGGGCGGTGTGTAC-3'	This paper	NA
18S RNA -1832 m6A- Forward primer<br>5’-CCGATTGGATGGTTTAGTG-3'	This paper	NA
18S RNA -1832 m6A- Reverse primer<br>5’-TAATGATCCTTCCGCAGG-3'	This paper	NA

References

1. Peltonen K, Colis L, Liu H, Trivedi R, Moubarek MS, Moore HM, et al. A targeting modality for destruction of RNA polymerase I that possesses anticancer activity. Cancer Cell. 2014;25(1):77-90.
2. Spandidos A, Wang X, Wang H, Seed B. PrimerBank: a resource of human and mouse PCR primer pairs for gene expression detection and quantification. Nucleic Acids Res. 2010;38(Database issue):D792-9.
3. Kwon I, Xiang S, Kato M, Wu L, Theodoropoulos P, Wang T, et al. Poly-dipeptides encoded by the C9orf72 repeats bind nucleoli, impede RNA biogenesis, and kill cells. Science. 2014;345(6201):1139-45.
