# Supplementary material for: TRMT112 drives a tumor growth and metastasis-promoting program in triple-negative breast cancer
Source: Cell Death Differ. 2026 Jan 8;33(6):1192–202. doi: 10.1038/s41418-025-01643-z (PMC13246786; doi:10.1038/s41418-025-01643-z)
Supplement: Supplementary file 4 — Related Manuscript File [file 41418_2025_1643_MOESM4_ESM.pptx]

## Slide 1
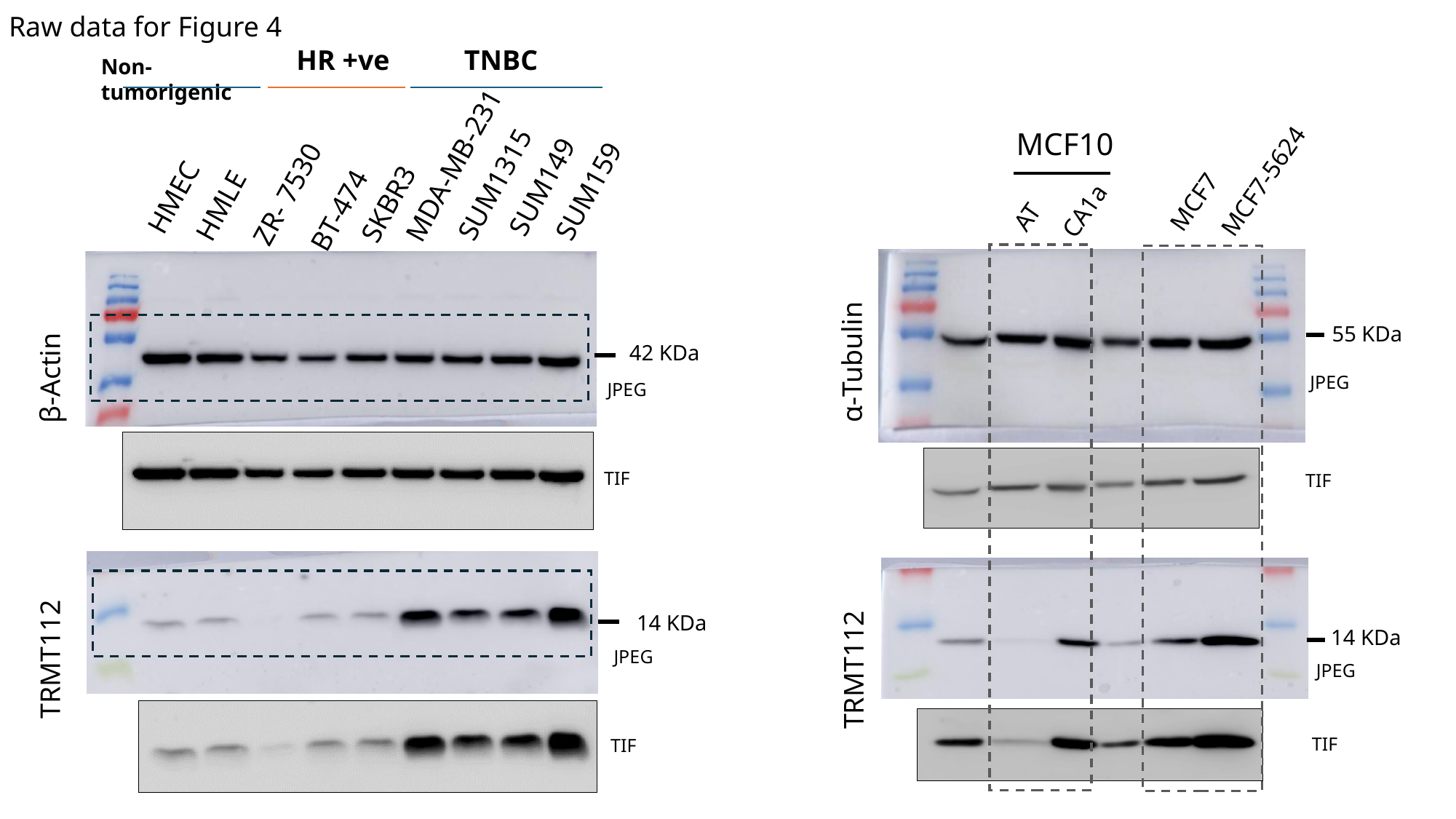

Raw data for Figure 4
HR +ve
TNBC
Non-tumorigenic
MDA-MB-231
SUM1315
SUM149
SUM159
ZR- 7530
HMEC
SKBR3
HMLE
BT-474
42 KDa
β-Actin
JPEG
TIF
14 KDa
TRMT112
JPEG
TIF
MCF10
MCF7-5624
MCF7
CA1a
AT
55 KDa
α-Tubulin
JPEG
TIF
14 KDa
TRMT112
JPEG
TIF

## Slide 2
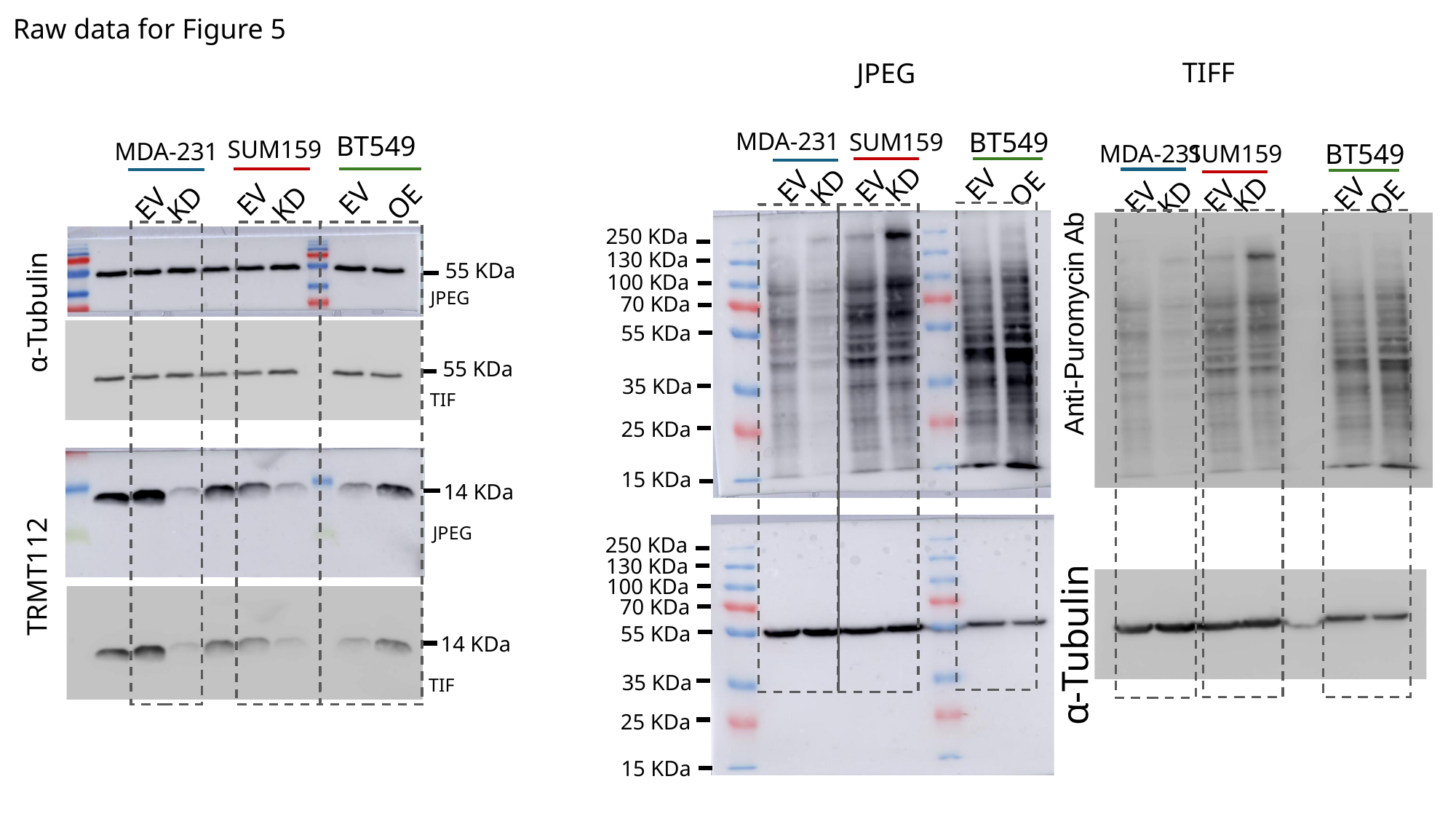

Raw data for Figure 5
TIFF
JPEG
BT549
MDA-231
SUM159
BT549
SUM159
MDA-231
KD
EV
EV
KD
EV
OE
EV
KD
EV
EV
KD
OE
250 KDa
130 KDa
100 KDa
70 KDa
Anti-Puromycin Ab
55 KDa
35 KDa
25 KDa
15 KDa
250 KDa
130 KDa
100 KDa
70 KDa
55 KDa
35 KDa
25 KDa
15 KDa
α-Tubulin
BT549
SUM159
MDA-231
EV
EV
OE
EV
KD
KD
55 KDa
JPEG
α-Tubulin
55 KDa
TIF
14 KDa
JPEG
TRMT112
14 KDa
TIF

## Slide 3
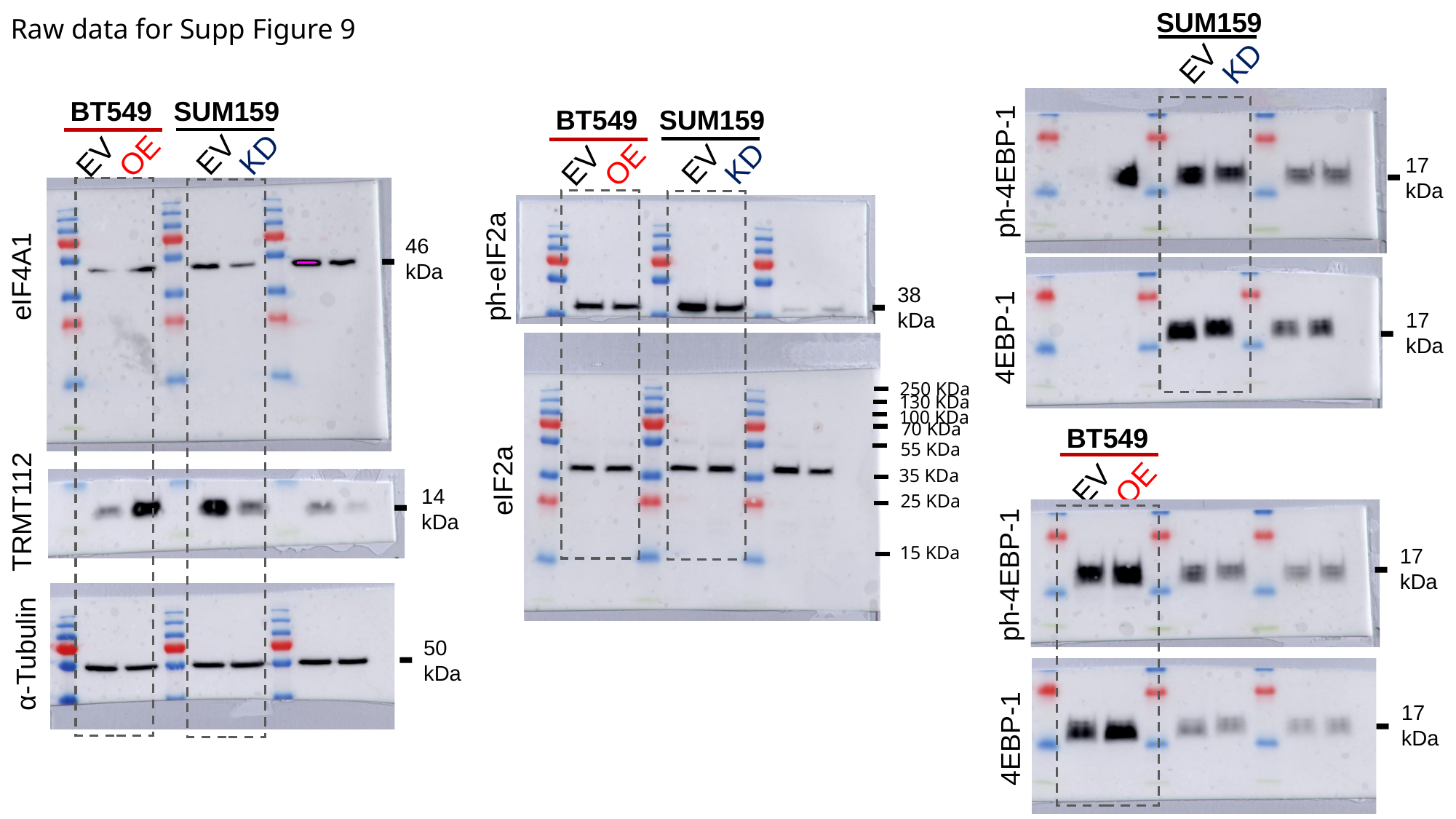

SUM159
KD
EV
-
17
kDa
ph-4EBP-1
-
17
kDa
4EBP-1
Raw data for Supp Figure 9
BT549
SUM159
KD
EV
OE
EV
46
kDa
eIF4A1
-
14
kDa
TRMT112
-
50
kDa
α-Tubulin
-
BT549
SUM159
KD
EV
OE
EV
-
38
kDa
ph-eIF2a
eIF2a
250 KDa
130 KDa
100 KDa
70 KDa
55 KDa
35 KDa
25 KDa
15 KDa
BT549
OE
EV
-
17
kDa
ph-4EBP-1
-
17
kDa
4EBP-1
